# Patients with Bacterial Sepsis Are Heterogeneous with Regard to Their Systemic Lipidomic Profiles

**DOI:** 10.3390/metabo13010052

**Published:** 2022-12-29

**Authors:** Knut Anders Mosevoll, Bent Are Hansen, Ingunn Margareetta Gundersen, Håkon Reikvam, Øyvind Bruserud, Øystein Bruserud, Øystein Wendelbo

**Affiliations:** 1 Section for Infectious Diseases, Department of Medicine, Haukeland University Hospital, 5021 Bergen, Norway; 2 Section for Infectious Diseases, Department of Clinical Research, University of Bergen, 5021 Bergen, Norway; 3 Department of Medicine, Central Hospital for Sogn and Fjordane, 6812 Førde, Norway; 4 Section for Hematology, Department of Medicine, Haukeland University Hospital, 5021 Bergen, Norway; 5 Leukemia Research Group, Department of Clinical Science, University of Bergen, 5021 Bergen, Norway; 6 Department for Anesthesiology and Intensive Care, Haukeland University Hospital, 5021 Bergen, Norway; 7 Faculty of Health, VID Specialized University, Ulriksdal 10, 5009 Bergen, Norway

**Keywords:** sepsis, bacteria, lipid, metabolism, metabolomics profile, patient heterogeneity

## Abstract

Sepsis is defined as life-threatening organ dysfunction caused by a dysregulated host response to infection. In the present study, we investigated the systemic/serum lipidomic profile at the time of hospital admission for patients with bacterial sepsis. The study included 60 patients; 35 patients fulfilled the most recent 2016 Sepsis-3 criteria (referred to as Sepsis-3) whereas the remaining 25 patients had sepsis only according to the previous Sepsis-2 definition and could be classified as having Systemic Inflammatory Response Syndrome (SIRS). A total of 966 lipid metabolites were identified. Patients fulfilling the Sepsis-3 criteria differed from the Sepsis-2 patients with regard to only 15 lipid metabolites, and especially sphingolipids metabolism differed between these patient subsets. A total of only 43 metabolites differed between patients with and without bacteremia, including 12 lysophosphatidylcholines and 18 triacylglycerols (15 C18/C20 fatty acid metabolites decreased and three C14 myristate acid metabolites that were increased in bacteremia). Unsupervised hierarchical clustering analyses based on the identified sphingolipids, phosphatidylcholine and triacylglycerols showed that (i) the majority of Sepsis-3 patients differed from SIRS patients especially with regard to lysophosphatidylcholine levels; (ii) the minority of Sepsis-3 patients that clustered together with the majority of SIRS patients showed lower Sequential Organ Failure Assessment (SOFA) scores than the other Sepsis-3 patients; and (iii) the variation between the patients in the identified/altered sphingolipid and triacylglycerol metabolites further increased the heterogeneity of Sepsis-3 patients with regard to their systemic lipidomic profile at the time of diagnosis. To conclude, patients fulfilling the Sepsis-3 criteria differ with regard to their metabolic profile, and this variation depends on disease severity.

## 1. Introduction

Sepsis is a common condition that is associated with high mortality and, for many of the survivors, long-term morbidity [1]. It is defined as life-threatening organ dysfunction caused by a dysregulated host response to infection, and the organ dysfunction can then be defined and classified according to the Sequential Organ Failure Assessment (SOFA) [2,3]. A subset of sepsis patients has or develops septic shock characterized by profound circulatory/cellular/metabolic dysfunctions and higher mortality; additional clinical characteristics of these patients are vasopressor requirement and high serum lactate levels in the absence of hypovolemia [1,2,3].

Sepsis is associated with metabolic dysfunctions; at the cellular level these abnormalities include mitochondrial dysfunction with altered energy metabolism and production of reactive oxygen species [3]. This metabolic dysfunction is possibly important for the development of organ failures and seems to involve lipid metabolism. Lipids are important for several cellular functions. First, they are essential parts of cellular membranes, and thereby they become important for cellular structure, formation of cellular compartments and initiation of intracellular signaling by various cell surface receptors [4,5]. Second, various lipids can serve as extracellular mediators that bind to specific cell surface receptors as a part of intercellular communication, or they can function as intracellular mediators that contribute to the regulation of fundamental cellular functions [6,7,8,9,10]. Finally, lipids are important nutrients, and thereby they become important for cellular energy metabolism [11,12].

A severe infection will usually initially cause an acute phase reaction, i.e., a reaction mediated by proinflammatory cytokines characterized by altered levels of acute phase proteins (e.g., increased C-reactive protein (CRP) levels and decreased albumin levels) due to an effect of the infection on distant organs, especially the liver, where many acute phase proteins are synthesized [13]. According to the definition and staging of sepsis, this is also a systemic reaction/complication in distant organs to an (initially) localized infection [1,2,3]. Sepsis-associated systemic metabolic modulations detected in serum/plasma may thus reflect an effect of local infection and inflammation on distant organs similar to the acute phase reaction [13,14], development of organ dysfunctions [3] and/or the metabolic status/requirements of inflammatory cells at the site of infection. In this context, we therefore investigated the lipidomic profiles of patients with bacterial sepsis at the time of hospital admission, prior to the start of antibiotic and supportive treatment, and we had a main focus on structurally defined lipid metabolite subsets rather than on the levels of individual metabolites. 

## 2. Materials and Methods

### 2.1. Patients

We previously performed a prospective study at Haukeland University Hospital which is a tertiary hospital in western Norway that also functions as a local emergency hospital for approximately 300,000 inhabitants [15]. Adult patients admitted with sepsis to the emergency department between December 2012 and 2014 were included. A total of 164 consecutive patients were admitted with clinical sepsis according to the Sepsis-2 criteria (definitions in [16]; more detailed review and discussion [17], but only 80 of them were immunocompetent patients with a later documented bacterial infection where 65% fulfilled the Sepsis-3 criteria and the others only fulfilled the Sepsis-2 criteria [1,2,3]. Patients with viral and parasitic infections, those without proven infections, immunocompromised patients (known congenital or acquired immunodeficiency) as well as patients receiving immunosuppressive/cytotoxic treatment were excluded. All included patients provided written informed consent for study participation. The study was approved by the regional Ethics Committee (REK Vest Norway, numbers 214849), and conducted in accordance with the Declaration of Helsinki.

Our present study included 60 patients with bacterial sepsis according to Sepsis-2 criteria, 30 patients had infections with Gram-positive and 30 patients with Gram-negative bacteria. We included 30 patients in each of these two groups to allow reliable bioinformatical comparisons between the two groups. The selection of patients can be seen from Tables S3 and S4 and from Figure S1. Our present study thus included only 60 out of the 80 patients in the original study that fulfilled the Sepsis-2 score-based criteria [15]. The 20 excluded patients were: (i) five patients with mixed infections, i.e., evidence for two infecting bacteria; (ii) eight patients with exceptional bacterial etiology, i.e., one patient each with *Enterobacter cloacae*, *Acinubaculum schalii*, *Bacteroides fragilis*, *Fusobacterium necroforum*, *Kingella kingae*, *Neiseria meningitidis*, *Klebsiella pneumoniae* and *Clostridium* infection; (iii) six (randomly selected from eight) patients where the bacterial diagnosis was based on detection of bacterial antigen alone but with no bacterial growth for any patient samples; and (iv) one randomly selected patient with Gram-negative infection (*Escherichia coli* blood culture) that was removed to obtain two equal groups with 30 patients with Gram-negative and Gram-positive infection, respectively. Thus, 19 of the 80 Sepsis-2 patients were left out to have more homogeneous but still relevant patients for the Gram-negative/positive comparison, but the 60 included patients also allowed reliable comparisons of patients with and without bacteremia (30 versus 30 patients) and patients only with severe inflammatory response syndrome (25 SIRS patients only fulfilling the Sepsis-2 criteria as described below) versus patients with organ dysfunction/failure (35 patients also fulfilling the Sepsis-3 criteria).

Fifteen of our patients with Gram-positive and 15 of our patients with Gram-negative infection had bacteremia, and these 30 patients were significantly older than the patients without blood stream infection (median age 69.5 versus 60.0 years, Mann–Whitney U test *p* = 0.035), had a significantly lower diastolic blood pressure (50 versus 67 mmHg, *p* = 0.003) at admittance as well as higher frequencies of both renal failure (77 versus 40%, Fisher’s exact test *p* = 0.004), respiratory failure (43 versus 13%, *p* = 0.030) and thrombocytopenia (33 versus 10%, *p* = 0.028) (Table S1). A major difference between patients with Gram-negative and Gram-positive infections was the site of the infection (Table 1); the large majority of patients with Gram-negative infections had urinary tract infections whereas most patients with Gram-positive infections had respiratory or soft tissue infections. Patients with Gram-negative infections also had higher age (median 73.5 versus 60 years, *p* = 0.043) and higher frequencies of patients with respiratory failure (77 versus 40%, *p* = 0.004) and central nervous system failure (23 versus 3%, *p* = 0.023) (Table S2). Finally, only four of the patients had a cause of sepsis that needed to be considered for surgical intervention, all other patients were treated in the Department of Medicine or Department of Pulmonary Diseases possibly in collaboration with the Intensive Care Unit.

We classified our patients according to the Sepsis-3 [1] and Sepsis-2 definitions [16,17]. All the 60 patients included in the present study fulfilled the Sepsis-2 criteria (i.e., based on the Systemic Inflammatory Response Syndrome, SIRS) whereas only 35 patients fulfilled the Sepsis-3 criteria (i.e., based on Sequential Organ Failure Assessment, SOFA). Furthermore, the presence of organ failure at the time of hospital admission according to the Sepsis-3 definition (35 patients out of the 60 patients) and the detection of bacteremia (30 patients) showed a highly significant association (Fisher’s test, *p* = 0.0002). Finally, for the 35 patients with Sepsis-3/organ dysfunction/failure Gram-negative and Gram-positive infections did not differ significantly with regard to overall SOFA classification or bacteremia (data not shown).

### 2.2. Lipidomic Analyses

Serum samples were derived at the time of admittance to the hospital and were stored frozen at −80 °C until analyzed. Serum was prepared within two hours after sampling, immediately aliquoted and stored frozen at −70 °C. Repeated thawing and freezing of samples was avoided.

Lipidomic analyses were performed by using the Complex Lipid Analysis platform of Metabolon (Morrisville, NC, USA) as described in detail previously [18]. A more detailed description of the procedures used by Metabolon is also given in the Supplementary Information (pages 18–21; Description of methodological strategies used by Metabolon for lipidomic analysis of patient samples).

### 2.3. Statistical and Bioinformatical Analyses

Following log transformation and imputation of missing values with the minimum observed value for each metabolite, ANOVA contrast was used to identify metabolites that differed significantly between the various patient subsets. Fisher’s exact test was used for comparison of categorized data, the Mann–Whitney U-test was used for comparison of continuous data, the Binomial test for single proportion was also used and the Benjamini–Hochberg analysis as a correction for analysis of multiple single metabolites.

The enrichment of a specific metabolic pathway when analyzing the uncorrected *p*-values can be seen from a 2 times 2 table including: (i) the number of statistically significant metabolites and the number of nonsignificant metabolites for the specific metabolic pathway to be considered; versus (ii) the number of significant and non-significant metabolites among all other metabolites not included in this particular pathway. We used the Fisher’s exact test for statistical evaluation of categorized data.

We used hierarchical clustering analyses for bioinformatical analysis of patient heterogeneity [15]. Briefly, these were performed using the J-Express software (MolMine AS, Bergen, Norway). Mediator values were initially log10 and Z-transformed for standardization of the data before clustering. Our analyses were based on the combination of Euclidean distance and complete linkage because this methodological approach gave the best homology between mediator concentrations and the most compact clusters.

## 3. Results

### 3.1. Identified Lipid Metabolites in Serum Samples Derived from Sepsis Patients

We detected a total of 966 lipid biochemicals in our 60 patient serum samples. The distribution of these 966 metabolites into various metabolic pathways/classes is presented in Table 1 (the two left columns). The metabolites could be grouped into the three main classes phospholipids (277 biochemicals), sphingolipids (61 biochemicals) and neutral complex lipids (628 biochemicals). The largest subclass was triacylglycerols that included more than half of all identified lipid metabolites (517 biochemicals).

The metabolites showed a wide variation between various patient subsets, as exemplified by the data presented in Figure S2. Furthermore, the patient heterogeneity was also reflected in Principal component analyses based on the overall metabolite levels; these analyses did not show any clear separation between Sepsis-3 and Sepsis-2 patients or patients with and without bacteremia (Figure S3).

### 3.2. Systemic Lipidolomic Profiles in Patients with Bacterial Sepsis: Lipidomic Characteristics Associated with Organ Failure (Sepsis-3 versus Sepsis-2), Bacteremia and Gram-Positive vs. Gram-Negative Infections

We identified lipid metabolites that differed significantly when comparing (i) patients with and without organ failure (patients fulfilling Sepsis-3 criteria versus patients only fulfilling Sepsis-2 criteria; 15 metabolites), (ii) patients with and without bacteremia (43 metabolites identified), and (iii) patients with Gram-negative versus Gram-positive infections—18 metabolites). These results are summarized in Table 2. We identified a total of 966 metabolites, and a significant difference was defined as an uncorrected *p*-value < 0.05. According to this definition we would expect to detect up to 58 significantly differing metabolites (i.e., one out of 20) by coincidence for statistical comparisons based on all the 966 metabolites, and we would, in addition, expect a random distribution of such coincidental metabolites among the different structure-based metabolic subsets/subclasses. Furthermore, if the identification of significantly differing metabolites was due to coincidence alone, we would, in addition, expect an equal number of metabolites showing increased and decreased levels among the various subsets/classes of significant metabolites. Even though each of the three comparisons described above in Table 2 identified less than 58 metabolites, for several reasons we regard the observed differences for three metabolic subclasses to (mainly) reflect true biological differences and not coincidence (see Table 2). These three metabolic subclasses either included at least 10% significantly differing metabolites (i.e., Sepsis-3 versus Sepsis-2, Sphingolipids; without versus with bacteremia, lysophosphatidylcholines) or included more than 10 single metabolites (without versus with bacteremia, triacylglycerols):

- Patients with organ failure (i.e., fulfilling the Sepsis-3 definition) showed altered levels of eight out of 62 sphingolipid metabolites (Table 2, Tables S5 and S6) whereas only seven out of the 904 non-sphingolipid metabolites differed between these two patient subsets; this is a statistically significant difference in the frequency of altered metabolites (i.e., relative metabolic pathway enrichment as described in the Materials and Methods Section 2.3) when comparing sphingolipid and non-sphingolipid metabolites (Fisher’s exact test, *p* < 0.00001). Furthermore, all dihydroceramides showed increased levels whereas the other sphingolipids showed increased levels; and these two metabolite subsets also showed a covariation in the later clustering analyses (see Section 3.5). Finally, the serum concentrations presented in Table S6 also illustrate that even for individual metabolites showing statistically significant differences between Sepsis-3 and Sepsis-2 patients, there was a large overlap in the serum levels. There was also a wide variation between the 15 significant metabolites with regard to their serum levels.
- Patients with bacteremia showed significantly altered levels of 12 out of 18 lysophosphatidylcholine metabolites (Table 2 and Table S7); this frequency of altered metabolites is significantly different (i.e., representing a pathway enrichment, see Material and Methods Section 2.3) from the frequency of significantly altered metabolites among the 948 non-lysophosphatidylcholine metabolites (31 out of 948 metabolites, Fisher’s exact test, *p* < 0.00001). Furthermore, all 12 metabolites showed decreased levels in patients with bacteremia, and this is also significantly different from the equal distribution of increased and decreased levels that would be expected if the cause was coincidence alone (Binomial test for single proportion, *p* = 0.0168).
- Patients with bacteremia constituted the only patient subset that showed altered levels for a relatively large number of triacylglycerols (Table 2 and Table S8; 20 metabolites including 18 triacylglycerols), but this frequency by itself is not significantly different from what would be expected by coincidence alone (expected frequency 26 out of 517 acylglycerol metabolites). However, if these triacylglycerol-associated differences were due to coincidence alone we would expect an equal number of increased and decreased metabolites, but 15 of these 18 metabolites showed decreased levels in bacteremia and this is significantly different from the equal distribution that would be expected by coincidence (Binomial test for single proportion, *p* = 0.001). Furthermore, there is also a structural difference between the 15 decreased and the three increased triacylglycerols; all the decreased metabolites involved C18/20 fatty acids whereas the three increased metabolites involved C14 fatty acids (see Section 3.5 for details).
- Several metabolites showed a *p*-value < 0.001; this was true for (i) one sphingolipid metabolite associated with Sepsis-3/organ failure, (ii) four metabolites showing increased levels in patients with bacteremia (two phosphatidylethanolamines, one lysophosphatidylcholine and one triacylglycerol), and (iii) three metabolites that differed significantly between Gram-positive and Gram-negative infections (two phosphatidylethanolamines and one sphingolipid) (Table S2).

Taken together these results suggest that patients with bacterial sepsis are heterogeneous with regard to their systemic lipidomic profile. These differences are observed mainly when considering the overall results for the three defined metabolite subsets/structural subclasses (analyses of pathway enrichment or binomial distribution; *p*-values would still be significant after Bonferroni correction for these six different comparisons), whereas relatively few single metabolites showed highly significant differences. Organ failure seems to be associated with altered sphingomyelin metabolism whereas patients with bacteremia show differences in lysophosphatidylcholine metabolites and a minor difference in triacylglycerol metabolism. In contrast, when comparing patients with Gram-positive and Gram-negative sepsis, we could only identify a relatively small and heterogeneous subset of significantly differing metabolites; this is consistent with a major influence of coincidence. Furthermore, identification of organ failure (fulfilling Sepsis-3 criteria) and the presence of bacteremia were significantly correlated, and both these parameters are this associated with an adverse prognosis for patients with bacterial sepsis. Therefore, to reduce the impact of coincidence in our later bioinformatical/clustering analyses (see Section 3.5, Section 3.6 and Section 3.7) of clinically relevant patient heterogeneity, we based all the clustering analyses only on the (i) lysophosphatidylcholine metabolites and/or the triacylglycerol metabolites identified in the comparison of patients with and without bacteremia; and/or (ii) the sphingolipid metabolites identified in the Sepsis-3/sepsis-2 comparison. All the individual metabolites in these three groups differed significantly in the statistical analyses as described above, and the overall characteristics of each of these three groups (including the covariation in our clustering analyses, see Section 3.5, Section 3.6 and Section 3.7) could not be explained by coincidence alone.

### 3.3. Differences in the Total Serum Levels of Various Lipids: The Impact of Organ Failure, Bacteremia and Bacterial Etiology

We also compared the total metabolite levels for all the 14 metabolite subclasses that are listed in Table 2, for bacterial sepsis patients (i) with and without organ failure according to the Sepsis-3/Sepsis-2 definitions; (ii) with and without bacteremia at the time of admission to the hospital, and (iii) patients with Gram-positive and Gram-negative infections. Only two of the 14 metabolite subclasses listed in Table 2 showed significant differences. First, patients with bacteremia showed increased levels of total lysophosphatidylcholines (one-way Anova, *p* = 0.024), whereas patients with Sepsis-3/organ failure showed decreased levels of sphingomyelins (*p* = 0.0418). Although these differences reached only borderline significance, they support the observations and our conclusions based on the analyses of individual metabolite subsets (see last chapter in Section 3.2).

### 3.4. Identification of a Phosphatydylethanolamine Metabolite That Reached Statistical Significance Also after Benjamini–Hochberg Analysis

Our identification of differing lipid metabolite subsets was based on uncorrected *p*-values, and this is the reason for our main focus on metabolite subsets/groups throughout the results presentation rather than individual metabolites. However, to further investigate the possible importance of individual metabolites we did Benjamini–Hochberg analyses. Only the phosphatidylethanolamine metabolite PE(O-18:0/18:0) remained significant after this correction, and this was seen both for the Sepsis-3/Sepsis-2 comparison and for the comparison of patients with and without bacteremia (Table S9). However, we would emphasize that this metabolite reached detectable levels (≥0.28 μM) only for five patients with Sepsis-3-bacteremia.

To conclude, the analyses show that these subsets of patients with bacterial sepsis do not show extensive metabolic differences that are reflected in significantly altered levels of individual single metabolites, when the patients are compared early during the disease course at the time of hospital admission/diagnosis. However, when considering the overall results for certain well-defined structural metabolic subclasses/pathways we observe differences (i.e., metabolic heterogeneity early during the disease) for a limited number of subclasses (lysophosphatidylcholines, sphingolipids and triacyglycerol subsets, see Section 3.2 and Table 2).

### 3.5. Lipidomic Profiles Associated with Sepsis-3 Classification and Detection of Bacteremia: Analyses Based on the Overall Profile of Significantly Altered Metabolites

We performed unsupervised hierarchical clustering analyses based on all the significantly differing metabolites expressed in at least 10 patients when comparing (i) patients fulfilling the criteria for Sepsis-3 versus patients that only fulfilled the Sepsis-2 criteria (13 out of the 15 metabolites, see Table S5), and (ii) patients with and without bacteremia (38 out of the 43 metabolites, see Table S7). We did not perform a similar analysis based on patients with Gram-negative vs. Gram-positive comparison because we then identified only 18 and relatively heterogeneous metabolites without significantly differing frequencies for any subset/groups of metabolites (see Section 3.2, Table 2 and Table S8); these observations suggest that metabolites identified by coincidence represent a major part among these 18 identified metabolites and for this reason these metabolites were not further analyzed by unsupervised hierarchical clustering analysis.

**Figure 1:**
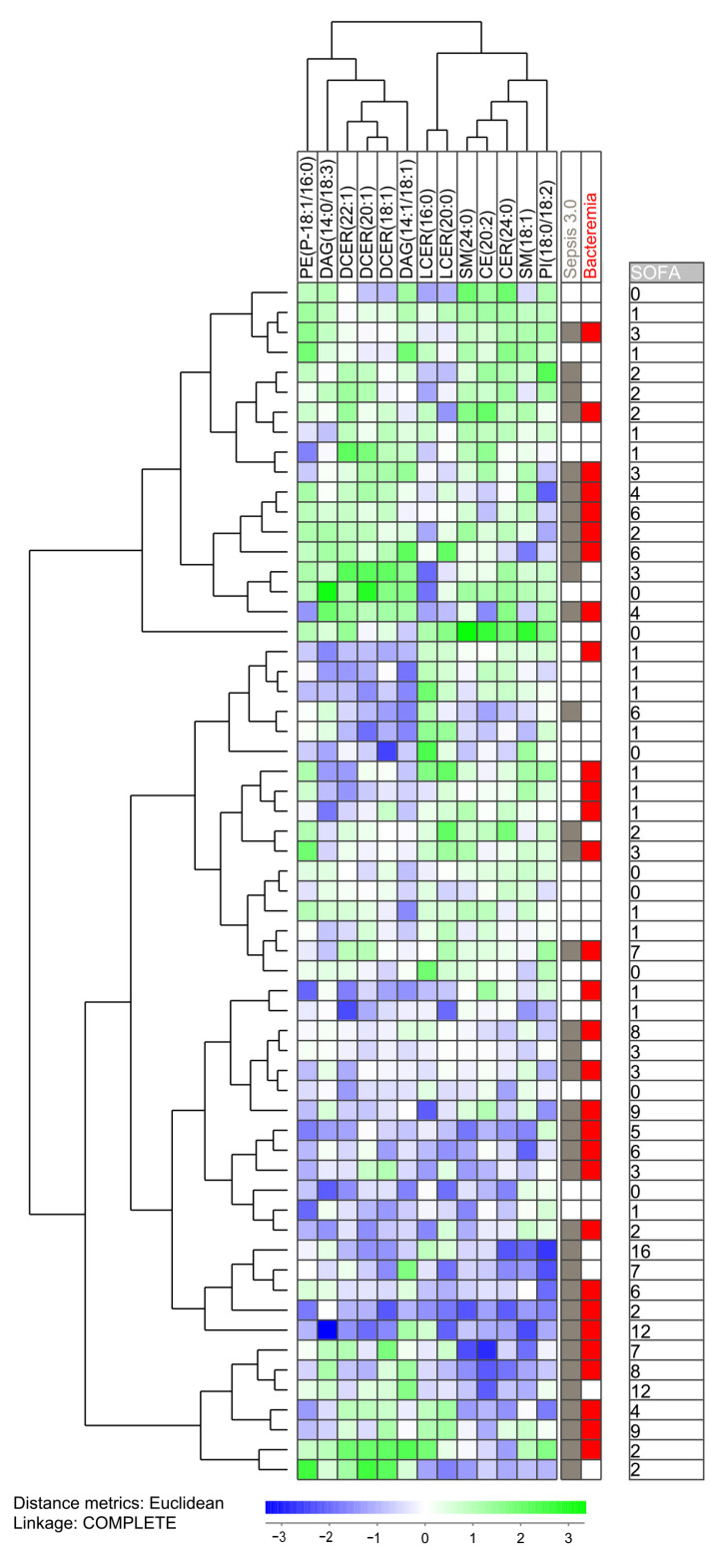
Subclassification of sepsis patients based on the 13 metabolites (mainly sphingolipids) that differed significantly when comparing patients fulfilling the Sepsis-3 criteria versus patients that only fulfilled the Sepsis-2 criteria. The analysis was based on the 13 metabolites (i) showing detectable levels for at least 10 patients and (ii) showing a statistically significant difference when comparing the two patient subsets. All 60 patients were included in the analysis. The characteristics of each individual patient (organ failure, bacteremia, total SOFA score) is indicated to the right in the figure.

The clustering analysis based on the 13 Sepsis-3 associated metabolites identified two main subsets: a smaller main upper subset including only 18 patients and a larger main subset including 42 patients (Figure 1). Most of the Sepsis-2 patients clustered close to each other either in the upper or lower part of the clustering. A separate subcluster of the lower main cluster then included a total of 17 patients with only four of them fulfilling the Sepsis-3 criteria; this is significantly different from the other patients (31 Sepsis-3 patients out of 43 patients; Fisher’s exact test, *p* = 0.001). Taken together, these observations suggest that even though these 13 lipid biochemicals differ between Sepsis-3 and Sepsis-2 patients, Sepsis-3 patients are heterogeneous with regard to this sphingomyelin profile and a minority of Sepsis-3 patients shows a profile that is most common for patients only fulfilling Sepsis-2 criteria. Finally, two main metabolite clusters could be identified (see the top of Figure 1), and metabolites belonging to the same sphingolipid subset clustered close to each other in the same main subcluster, e.g., dihydroceramides and lactocylceramides clustered close to each other in two different metabolite subclusters. Thus, there is a covariation between related sphingolipids.

We also did a clustering analysis based on 38 of the 43 metabolites that differed significantly between patients with and without bacteremia (i.e., five metabolites excluded because they were detected in less than 10 patients) (Table 2 and Table S7; Figure S4). We detected two main patient clusters, and the larger upper main cluster (41 patients) could be further divided into two subcluster where the upper subcluster included 19 patients and only four of them fulfilled the Sepsis-3 criteria (Figure S4). This low frequency of Sepsis-3 patients is significantly different from the other 41 patients (4/19 versus 31/41, Fisher’s exact test, *p* = 0.0001). Furthermore, the lipid metabolites formed two main clusters (see the top of Figure S4). All the lysophosphatidylcholines clustered together within the left main cluster, i.e., there is a covariation between the different lysophosphatidylcholines when comparing individual patients. This covariation is reflected in the metabolite clustering (top of Figure S4) and the covariation/clustering cannot be explained by coincidence when these metabolites were identified as described in Table 2/Section 3.2. Furthermore, all the 18 triacylglycerols were localized in the right main metabolic cluster; all the C18/C20 triacylglycerols then clustered together in the largest subcluster whereas the three C14 triacylglycerols clustered together in the right subcluster. These covariations/coclusterings cannot be explained by coincidence either. A similar conclusion as for Sepsis-3 associated metabolites is justified: (i) Sepsis-3 patients are heterogeneous with regard to this lipid metabolite profile and a subset of Sepsis-3 patients show a profile that is more common for patients only fulfilling Sepsis-2 criteria; and (ii) lipid biochemical belonging to the same subclasses (i.e., lysophosphatidylcholines, C18/C20 and C14 triacylglycerols) show strong covariations that cannot be explained by coincidence.

### 3.6. The Metabolic Heterogeneity of Sepsis-3 Patients: A Comparison of the Lysophosphatidylcholine Profiles for Sepsis-3 Patients and Patients Only Fulfilling the Sepsis-2 Criteria

**Figure 2:**
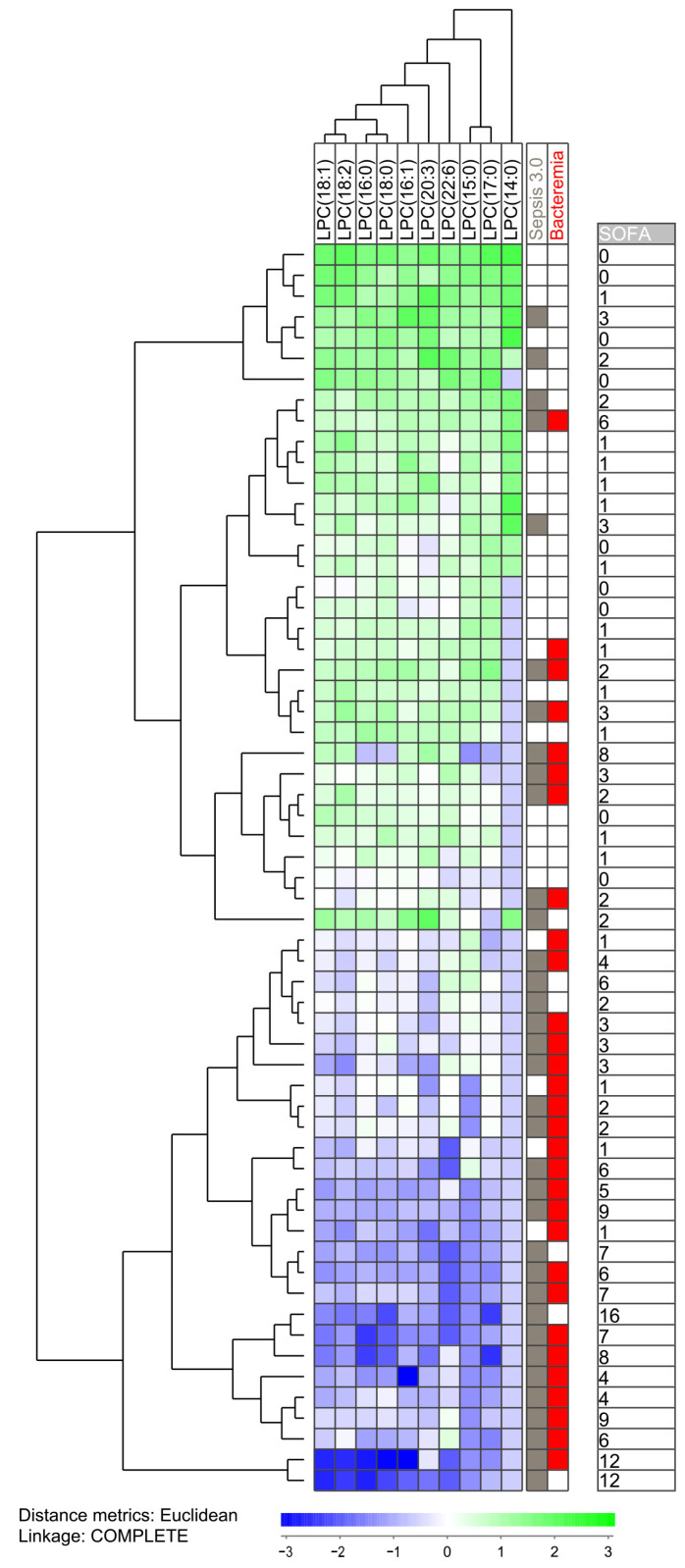
Subclassification of sepsis patients based on their lysophosphatidylcholine profile. The analysis was based on the lysophosphatidylcholines (LPC) (i) showing detectable levels for at least 10 patients and (ii) showing a statistically significant difference when comparing patients with and without bacteremia. All 60 patients were included in the analysis. The characteristics of each individual patient (organ failure, bacteremia, total SOFA score) are indicated to the right in the figure.

Our initial analyses identified three groups of lipid metabolites that differed significantly between either Sepsis-3 versus Sepsis-2 patients or between patients with and without bacteremia, i.e., eight sphingolipids, 12 lysophosphatidylcholines and 18 triacylglycerols (Table 2). To further characterize the heterogeneity of the sepsis patients we performed unsupervised hierarchical cluster analyses based on the lysophosphatidylcholines and identified two main patient clusters/subsets; the upper main cluster included 33 patients and the lower main cluster 27 patients (Figure 2). The fraction of patients fulfilling the Sepsis-3 criteria was significantly higher in the lower main cluster (23 out of 27 patients) compared with the upper main cluster (12 out of 33 patients; Fisher’s exact test, *p* = 0.0002). The lower main cluster also showed significantly higher total SOFA scores than the upper main cluster (Figure 2, Mann–Whitney U test, *p* < 0.0001).

We did a more detailed analysis the 35 patients that fulfilled the Sepsis-3 criteria by comparing the 12 Sepsis-3 patients in the upper main cluster in Figure 2 (i.e., patients showing similarities with Sepsis-2 patients) with the majority of Sepsis-3 patients in the lower main cluster (Table 3). This clustering analysis was based only on 10 phosphatidylcholine metabolites that differed significantly between Sepsis-3 and Sepsis-2 patients and could be detected for at least 10 patients (i.e., two metabolites left out). These two subsets of Sepsis-3 patients differed with regard to clinical severity/SOFA score that was higher for the lower main cluster (but with no significant difference with regard individual organ failure classification according to the Sepsis-3 guidelines), higher respiratory rate, higher serum CRP levels, higher serum creatinine levels and lower peripheral blood platelet counts. The two subsets did not differ with regard to frequency of Gram-positive/negative infections or localization of the infections. Thus, our overall results suggest that more severe sepsis is associated with a specific lysophosphatidylcholine profile, whereas Sepsis-3 patients with less severe disease show a profile similar to patients only fulfilling Sepsis-2 criteria.

These clustering analyses also showed that there is a in individual bacterial sepsis patients with regard to their systemic levels of lysophosphatidylcholines; individual patients show either generally high or low levels of lysophosphatidylcholines. This covariation is so strong that well-defined patient subsets could be identified based on the covariation of these metabolites, and this covariation cannot be explained by random selection/coincidence when identifying these metabolites (see Section 3.2 and Table 2 above).

### 3.7. The Metabolic Heterogeneity of Sepsis-3 Patients: A Comparison of Metabolic Profiles Based on Sphingolipids and Triacylglycerols

We also did two separate unsupervised hierarchical clustering analyses based on the eight sphingolipids (Figure S5) and the 18 triacylglycerols (Figure S6) identified by comparing Sepsis-3 versus Sepsis-2 patients and patients with/without bacteremia, respectively:

- Our clustering analysis based on the eight sphingolipid metabolites (Figure S5) identified two main clusters that did not differ with regard to frequency of Sepsis-2 patients. However, the lower main patient cluster included a subcluster/subset with only four Sepsis-3 patients among the 16 patients, and this is significantly different from the other patients (31 out of 44 patients, Fisher’s exact test, *p* = 0.0026). For the clustering of individual metabolites, we made a similar observation as for the previous clusterings (Figure 1, Figure 2 and Figure S3); metabolites belonging to the same sphingolipid subclass (the three dihydroceramides and the two lactosylceramides) showed covariations in individual patients; this was reflected in the formation of two separate metabolite subclusters, and also in the identification of distinct patient subsets/clusters based on the covariation/levels of these related metabolites.
- Our clustering analysis based on the 18 triacylglycerols (Figure S6) identified two main patient clusters that did not differ significantly with regard to frequency of Sepsis-3 patients and total SOFA score. Again, we observed a covariation between related metabolites that cannot be explained by coincidence. The metabolites showed two main clusters, the left main cluster included all 15 C18/C20 long-chain fatty acid metabolites whereas the right main clusters included the three C14 metabolites. Thus, the analysis confirmed that there is a metabolite covariation for triacylglycerol metabolites in individual patients, this cannot be explained by coincidence, and this covariation is the basis for identification of distinct patient subsets.

Based on the overall data from the clustering analyses of lysophosphatidylcholines (the previous Section 3.6), triacylglycerols and sphingolipids we conclude that (i) most Sepsis-3 patients show altered levels of phosphatidylcholines and this biological characteristic is associated with disease severity; (ii) the altered levels of sphingolipids and triacylglycerols do not show a similar strong association with Sepsis-3/disease severity; these two metabolite subsets rather contributes to an additional heterogeneity between individual Sepsis-3 patients.

## 4. Discussion

In our present study, we have investigated the systemic lipidomic profiles of patients with bacterial sepsis, and we observed that sepsis patients are characterized by a specific profile at the time of diagnosis. This profile may reflect an effect of local infection and inflammation on distant organs similar to the acute phase reaction [13,14], development of organ dysfunctions [3] and/or the metabolic status/requirements of the inflammatory cells at the site of infection. Thus, the molecular mechanisms behind this metabolic profile are probably diverse, and in our detailed analyses of the metabolomics data we focused on the Sepsis-3/Sepsis-2 comparison and bacteremia analyses (but not infection site and bacterial Gram-positive/negative classification) because both of these factors show highly significant associations to disease severity. Finally, we have a main focus on structurally defined lipid metabolite subclasses with statistical characteristics that could not be explained by coincidence, and not on individual metabolites.

As will be further discussed below, we did not detect extensive metabolic differences between clinically defined patient subsets when analyzing the systemic levels of single/individual metabolites early during the disease course at the time of diagnosis. However, based on studies of metabolic pathways (i.e., structurally based subclasses of metabolites) we observed differences in the three metabolic subsets lysophosphatidylcholines, sphingolipids and a subset of triacylglycerols (see Section 3.2 and Table 2). The differences for these three subsets could not be explained by coincidence alone based on pathway enrichment analyses and/or binomial analysis of the numbers of increased/decreased metabolites in the specific group. Our clustering analyses, in addition, showed extensive covariation in individual patients for the metabolites belonging to the same subclass, and this metabolic covariation was also the basis for identification of defined patient clusters with regard to metabolic profiles. Taken together these observations support the hypothesis that there are (minor) lipidomic differences between patients with bacterial sepsis already early during the disease course at the time of hospitalization/diagnosis. For these reasons our detailed analyses of the metabolomic profiles focused only on these three identified metabolic subclasses, i.e., only defined subsets of, but not all individual metabolites (see Table 2) identified by significant *p*-values before the Benjamini–Hochberg analysis. Future studies have to clarify whether further development/progression of these differences is seen for patients with later progression of their disease.

Our original study included 80 consecutive patients with bacterial sepsis that fulfilled the criteria of sepsis according to the Sepsis-2 criteria [16,17], and for the present study we included 60 of these patients based on the selection criteria, described in detail in Section 2.1. (see Tables S3 and S4, Figure 1). The 60 included and the 20 excluded patients of the original 80 consecutive patients are also listed and described in Tables S3 and S4. The 60 included patients are, in our opinion, representative of the main subsets of sepsis patients with regard to bacterial causes, and this is also supported in comparison to our present patients with patients included in large clinical studies [19,20,21,22].

The cellular lipidome is extremely extensive and theoretically consists of 9600 glycerophospholipids, more than 100,000 sphingolipids, thousands of mono-, di- and triglycerol variants and a wide range of fatty acid and sterol-based structures [23]. In the present study, we investigated the systemic serum levels of 966 different lipids in patients with sepsis. Even though the present study thus included only a small part of the total cellular lipidome, our results suggest that patients with untreated sepsis are heterogeneous with regard to their systemic lipidomic profile, especially systemic levels of lysophosphatidylcholines, sphingolipids and certain triacylglycerols.

Our samples were prepared according to a strictly standardized protocol for sampling, serum preparation and storage as described in the Materials and Methods section. However, samples were collected at admission to the hospital, and for this reason it was not possible to standardize the sampling with regard to food intake and diurnal variation. Despite this variation between patients, we could observe significant associations between metabolite levels and Sepsis-2 versus Sepsis-3 as well as bacteremia.

As stated above, the observed differences in serum levels of lysophosphatidylcholines, sphingolipids and certain triacylglycerols are probably caused by diverse mechanisms, including the local inflammation itself, the effect of the local inflammation on distant organs similar to the acute phase reaction and organ dysfunctions/failures due to sepsis [3,13,14]. However, immunoregulatory effects on these systemic levels due to their availability for the immunocompetent/inflammatory cells at the local inflammatory site may also be important. Cells from the innate immune system are the first to infiltrate at an inflammatory locus, and the effects of these lipids on such cells and especially the first arriving neutrophils may therefore be of particular importance. First, lysophosphatidylcholines can increase neutrophil adhesion, increase the expression of the CD11b adhesion molecule expression and increase the expression of pro-chemotactic receptors for formylated peptides [24]; local neutrophil infiltration is thereby increased [25]. These metabolites also have the potential to increase neutrophil degranulation [24], increase neutrophil bactericidal activity [26], and regulate extracellular trap formation [27]. Lysophosphatidylcholines can bind to the G-protein coupled G2A receptors and thereby initiate intracellular signaling and enhance the pro-inflammatory functions of both neutrophils [28] and monocytes/macrophages [29,30]. Second, several sphingolipids also function as immunoregulators and can modulate various neutrophil as well as monocyte functions including migration, phagocytosis and even survival, because sphingolipids inhibit neutrophil apoptosis [31,32,33,34,35,36]. However, the final effect on neutrophils seems to depend on the biological context and/or the dominating sphingolipid. These metabolites may also have indirect effects on neutrophils, especially neutrophil migration, through their activation of endothelial stress responses in sepsis patients. Both neutrophils and monocytes are important immunoregulatory cells with effects on other immunocompetent cells [37,38,39,40,41,42]. Taken together, these observations suggest that the lipidomic differences detected in our present study not only reflect the status of sepsis patients but probably also have an impact on the inflammatory response in these patients.

In our present study, we compared patients with sepsis as defined by the Sepsis-3 2016 criteria [1] compared with the previous Sepsis-2 criteria [16,17]. Sepsis-2 criteria defined sepsis as an inflammatory syndrome, whereas Sepsis-3 criteria in addition required the occurrence of organ failure. Previous clinical studies have also compared these two different criteria for sepsis and concluded that Sepsis-2 criteria defines a patient population with a larger subset of patients having a less severe disease compared with the Sepsis-3 criteria [43,44,45,46,47,48]; the patient subset “Sepsis-2 severe sepsis” and Sepsis-3 patients then seem to be comparable with regard to incidence, mortality and significant risk-adjusted improvements in mortality over time [43]. This is also consistent with our present patient subset analyses, i.e., as expected patient subset/clusters including a majority of patients that fulfilled the Sepsis-3 criteria showed generally higher total SOFA scores compared with other patient subsets/clusters.

The new Sepsis-3 definition defines sepsis as a life-threatening organ dysfunction caused by a dysregulated host response to infection [1,49,50,51]. Analyzing health record data on critically ill patients with suspected sepsis identified SOFA scoring as a predictor for poor outcome, but it is comprehensive and not suitable for use in emergency departments [51]. The simplified quick SOFA (qSOFA) score was therefore developed to facilitate easier identification of patients potentially at risk of dying from sepsis [49,50,51]. The qSOFA consists of three components (respiratory rate ≥ 22/min, change in metal state, systolic blood pressure ≤ 100 mmHg) all allocated at one point, and a qSOFA score ≥2 points indicates organ dysfunction. Sepsis-3 abandoned the use of SIRS criteria in identification of sepsis and eliminated the term severe sepsis. However, qSOFA scores show high specificity and poor sensitivity for early risk assessment and organ dysfunction [52,53], whereas the prognostic accuracy of SIRS for in-hospital mortality is low and the prognostic accuracy of the SOFA score is better [54,55]. Studies on the potential use of qSOFA as a screening tool for sepsis show contradictory results [56,57,58]. Sepsis-3 with qSOFA have been criticized [59,60], particularly because the use of qSOFA score risks missing early identification of sepsis when treatment is the most effective. Based on these studies, we decided to use the SOFA classification for our present patients.

We identified 15 significantly different metabolites when comparing patients fulfilling the Sepsis-3 criteria compared with patients only fulfilling the Sepsis-2 criteria, whereas 43 metabolites were significantly associated with the detection of bacteremia (Table 2, Tables S5 and S7). However, there was only a minor overlap between the significantly differing metabolites identified by these two comparisons, even though there was a significant association between Sepsis-3 and bacteremia (only 10 patients having bacteremia without fulfilling the Sepsis-3 criteria). Furthermore, organ failure was associated with altered sphingomyelin levels whereas bacteremia was associated with altered levels of lysophosphatidylcholine and triacylglycerols. Taken together, these observations suggest that organ failure and bacteremia influence the systemic lipidomic profiles through different mechanisms. This hypothesis is also consistent with our observations from the clustering analyses where especially the lysophosphatidylcholine metabolites associated with bacteremia were associated the strongest with Sepsis-3 and organ failure (Figure 2). In contrast, Sepsis-3 associated sphingolipids and bacteremia-associated triacylglycerols also differentiated between Sepsis-3 and Sepsis-2 patients, but these metabolites mainly seem to contribute to the heterogeneity among Sepsis-3 patients.

In our present study, bacteremia was associated with altered levels of lysophosphatidylcholine. Lysophosphatidylcholines are ligands for TLR2 and TLR4 and possibly also for the G2A and GPR4 receptors. Ligation of TLR2/4 will often increase the release of proinflammatory cytokines (e.g., by monocytes) [61,62]. Lysophosphatidylcholines modulate monocytes and T-cell chemotaxis through G2A ligation, whereas endothelial cells are modulated through the ligation of the GPR4 to express higher levels of adhesion molecules, microvessel permeability is altered and chemotaxis of immunocompetent/inflammatory cells is probably altered [63,64,65,66,67,68]. G2A also functions as a receptor for oxidized free fatty acids derived from linoleic and arachidonic acids [69]. GPR4 can bind both sphingosylphosphorylcholine and lysophosphatidylcholine and is thereby a regulator of vascular permeability and inflammation [66,70,71]. These lipids stimulate the release of pro-inflammatory cytokines, inhibit migration and proliferation of endothelial cells, activate and polarize macrophages towards the M1 phenotype, activate B cells and induce regulatory T cell differentiation [63,72,73]. Thus, lysophosphatidylcholines function as important regulators both for the innate and the adaptive immune system through direct and indirect effects on the immunocompetent cells.

Our study showed that organ failure (i.e., Sepsis-3 classification) was associated with altered levels of several sphingolipids, including sphingomyelin and several ceramides. Sphingomyelin is a component of the external leaflet of the plasma membrane and is also enriched in endosomes and the Golgi network; it interacts with cholesterol and this interaction modulates its biological functions [74,75,76,77]. Sphingomyelin and cholesterol are important for recruitment of (plasma) membrane proteins, and sphingomyelin is a precursor to ceramide [78,79]. Furthermore, there are functional/synthetic interactions between fatty acid synthesis, fatty acid uptake and sphingolipid metabolism [80,81,82]. Finally, ceramides are involved in these interactions that are important for the regulation of inflammation and the function of monocytes; i.e., it is involved in the regulation of innate immunity [83,84,85,86]. Both sphingomyelin [75] and ceramides [87,88] are thus involved in the regulation of innate immunity.

In our present study, we did not perform a detailed analysis of lipidomic profiles with regard to Gram-negative/positive infections and the locus of the bacterial infection. First, comparison of Gram-negative and Gram-positive infections identified relatively few and very heterogeneous metabolites that differed between these two groups. Second, this bacterial classification showed no associations with the SOFA score or results from clustering analyses. Third, the infectious sites were very diverse and our present study was, in our opinion, too small to analyze potential differences between infection sites and thereby between Gram positive and negative infections. For these reasons we focused our analyses on Sepsis-3 criteria and the associated detection of bacteremia.

Relatively few metabolites differed significantly between the various patient subsets that were compared in our present study (three different comparisons, see Table 2). Most of these individual metabolites did not reach significance when using the Benjamini–Hochberg procedure (Table S9). For this reason, our study is mainly based on the analysis of identified metabolite groups/subsets that showed statistically significant characteristics that could not be explained by coincidence (i.e., subset enrichment compared to the overall metabolites, increased/decreased ratios significantly different from binomial/coincidence distribution). For this reason, we emphasize that one should be very careful to focus on single metabolites identified in our present study but possibly with one exception; the Phosphatidylethanolamine/PE ether PE(O-18:0/18:0) remained significant both in the Sepsis-3/Sepsis-2 and the bacteremia comparison even after the Benjamini–Hochberg comparison (Table S9). However, even though this metabolite reached statistical significance, it should be emphasized that it reached detectable levels only for a small subset of Sepsis-3/bacteremia patients.

Seven other phosphatidylethanolamines reached statistical significance based on uncorrected *p*-values (Table 2, Tables S5 and S7), but this number was not sufficient to reach statistical significance in enrichment analysis based on Fisher’s exact test. Both lipophosphatidylcholines [89] and phosphatidylethanolamines [89,90,91,92,93] are involved in the regulation of inflammation. These lipid subsets are in addition substrates for phospholipase A2 IIA [94] that is an acute phase protein and a link between inflammation and lipid metabolism [95]. Systemic levels of phospholipase A2 are regarded as a potential biomarker in sepsis [96] and with a possible link to the immunoregulatory eicosanoid metabolism [97]. Thus, our present study together with these previous observations suggest that the possible importance and interactions between lysophosphatidylcholine, phosphatidylethanolamines, phospholipase 2 and eicosanoid metabolism should be further investigated in future clinical sepsis studies.

Several previous studies have investigated serum levels of lipoproteins and lipid classes in patients with sepsis; these results have been discussed in several recent reviews [98,99,100,101,102]. First, sepsis patients show decreased levels of High (HDL) and Low density (LDL) lipoproteins, and these levels are inversely correlated with the levels of pro-inflammatory cytokines. Second, levels of very low density lipoproteins (VLDL) can be increased in sepsis. Third, patients with sepsis also show altered triglyceride and fatty acid metabolism and altered levels of specific pro-resolving lipid mediators. These alterations of lipid metabolites will probably influence immunoregulation, endothelial protection and clearance of microbial lipids, and modulation of these mechanisms can at least partly explain the observed association between lipid levels (especially decreased HDL, HDL-cholesterol and cholesterol levels) and adverse prognosis in sepsis. In our present study, we could not detect such extensive metabolic differences between subsets of sepsis patients; although we detected abnormalities in fatty acid/triacylglycerol metabolism, we could not detect any major differences in cholesterol metabolism. The explanation for detection of less extensive lipidomic differences in our present study could be that we compare two groups that both have severe bacterial infections, sampling of patients early during the infection, and/or our selection of medical patients admitted to the hospital for acute illness.

Our study was based on a clinical study including consecutive adult patients requiring emergency admission to the hospital due to suspected sepsis [15]. All patients had bacterial infections and were relatively homogeneous with regard to bacterial etiology, and only four of them had a predisposition for sepsis that required surgical evaluation. In our opinion, our present study is representative of medical patients with sepsis. However, sepsis patients are heterogeneous, and this heterogeneity is probably an explanation for the conflicting results reported in previous metabolic studies of sepsis patients [103]. It should be emphasized that our results may not be representative for sepsis patients with uncommon bacterial etiologies, sepsis in patients requiring surgical intervention, sepsis as a complication after surgery or trauma, pediatric sepsis or sepsis in immunocompromised patients. Future studies have to clarify whether our present results are representative also of (any of) these patient groups. Finally, additional studies are needed to clarify the possible impact of the infection site on the lipidomic profile of sepsis patients, but the few and heterogeneous metabolites that differed between patients with Gram-negative (Table 1; many urinary tract infections) and Gram-positive infections (very few urinary tract infections) suggests that the impact of infection site on the lipidomic profiles of patients with bacterial sepsis may be limited.

## 5. Conclusions

Our present study shows that sepsis patients are heterogeneous with regard to their systemic lipidomic profiles, but our comparisons of Sepsis-3 versus Sepsis-2 patients and patients without and with bacteremia suggest that this heterogeneity is limited. Sepsis-3/organ failure is associated with altered levels of several sphingolipids compared with SIRS patients, whereas patients with bacteremia show altered levels of especially, phospholipids, but also certain long-chain triacylglycerols. Furthermore, patients fulfilling the Sepsis-3 criteria were heterogeneous with regard to their systemic profiles of sphingomyelins/lysophosphatidylcholines/triacylglycerols at the time of diagnosis, and this heterogeneity seems to be at least partly dependent on disease severity.

## Figures and Tables

**Table 1:** The site of infections for 60 sepsis patients with Gram-positive and Gram-negative infections included in the present study. The results are presented as the number and percentage of patients for each of these two patient subsets. Fisher’s exact test was used for the statistical analyses, and the corresponding *p*-values are presented in the right column (Ns, not significant).

Infection Site	Gram-Positive Infection (*n* = 30)		Gram-Negative Infection (*n* = 30)		*p*-Value
	Number	Percent	Number	Percent	
Urinary tract	2	7%	26	87%	<0.00001
Respiratory	8	26%	1	3%	0.0257
Soft tissue	11	37%	0	0	0.0003
CNS	3	10%	0	0	Ns
Endocarditis	5	17%	0	0	Ns
Other	1	3%	3	10%	Ns

**Table 2:** Lipidomic profiles in patients with bacterial sepsis; a summary of the overall results for all 60 patients and the 966 lipid biochemicals identified in the present study. The table presents the classification of various lipid metabolites, the total numbers of identified metabolites for each of the three main classes (written in bold) and their subclasses (Total number), number of differing metabolites when comparing patients with and without organ failure (i.e., Sepsis-3 vs. Sepsis-2 patients), patients with/without bacteremia and the comparison of patients with Gram-positive versus Gram-negative bacterial infections. The study included 60 patients (30 with Gram-positive and 30 with Gram-negative infections); 35 patients fulfilled the Sepsis-3 definition and 30 patients had bacteremia (14 with Gram-positive and 16 with Gram-negative bacteria).

Classification of the Identified Lipid Metabolites	Total Number	Sepsis-3 vs. Sepsis-2 Patients	Patients with vs. without Bacteremia	Patients with Gram-Negative vs. Gram-Positive Infections	Comment
**Phospholipids**	277				
Phosphatidylcholines	97	0	2	3	
Lysophosphatidylcholines	18	0	12	0	Patients with bacteremia showing increased overall frequency and increased levels for all 12 metabolites.
Phosphatidylethanolamines	123	1	4	9	
Lysophosphatidylethanolamines	15	1	1	1	
Phosphatidylinositols	24	1	0	1	
**Sphingolipids**	61	8	3	2	Increased overall frequency for patients with organ failures.
Ceramides	12	1	0	0	
Dihydroceramides	13	3	2	0	
Hexosylceramides	12	0	0	1	
Lactosylceramides	12	2	1	1	
Sphingomyelins	12	2	0	0	
**Neutral complex lipids**	628				
Cholesteryl Esters	26	0	0	0	
Diacylglycerols	58	4	2	4	
Triacylglycerols	518	0	18	0	Fifteen of the 18 altered metabolites in patients with bacteremia showed increased levels.
Monoacylglycerols	26	0	1	1	
**Total**	**966**	**15**	**43**	**21**	

**Table 3:** Subset classification of Sepsis-3 patients. A comparison of the lysophosphatidylcholine serum profile for the two Sepsis-3 patient subsets identified in the unsupervised hierarchical clustering analysis presented in Figure 2, i.e., the majority of Sepsis-3 patients localized in the lower main patient cluster and the minor subset localized in the upper main cluster. The results are presented as the median and variation range. The Mann–Whitney U test was used for the statistical analyses.

Parameter	Lower Main Cluster (*n* = 23)	Upper Main Cluster (*n* = 12)	*p*-Value
Total SOFA score	7 (2–16)	2.5 (2–8)	0.00382
Respiratory rate (per minute)	32 (15–60)	22 (14–30)	0.02852
Serum CRP (mg/L)	237 (57–457)	49 (4–423)	0.00062
Serum creatinine (mmol/L)	153 (54–475)	78 (65–163)	0.01352
Peripheral blood platelet count (× 10^9^/L)	193 (38–538)	260 (133–407)	0.0046

## Data Availability

The data presented in this study are available on request from the corresponding author. The data are not publicly available due to Norwegian legislation; making the lipidomic data available together with the necessary clinical information will require the names of collaborating scientists and then approval by the Regional Ethics Committee.

## Supplementary Materials

The following supporting information can be downloaded at: https://www.mdpi.com/article/10.3390/metabo13010052/s1, Table S1: Clinical characteristics of patients with and without bacteremia/blood stream infection (BSI); Table S2: Clinical and biological characteristics of patients with Gram-positive and Gram-negative infections; Table S3: The characteristics of individual patients included in the present study; Table S4: The selection of patients for the present study; Table S5: Differences of individual lipid metabolites between patients fulfilling the Sepsis-3 definition (PMID 26903338) versus patients only fulfilling the Sepsis-2 criteria (PMID 12682500, 12664219). Table S6. Differences of individual lipid metabolite concentrations between patients fulfilling the Sepsis-3 definition (PMID 26903338) versus patients only fulfilling the Sepsis-2 criteria (PMID 12682500, 12664219); Table S7: Differences of individual lipid metabolites between patients with and without bacteremia; Table S8: Differences of individual lipid metabolites between patients with Gram-negative and Gram-positive infection; Table S9. Benjamini-Hochberg analyses of significantly different metabolites identified by the comparison of Sepsis-3 versus Sepsis-2 patients (upper part), and patients with and without bacteremia (lower part); Figure S1: The selection of patients for the present study; Figure S2: The metabolic variation of sepsis patients; total lysophosphatidylcholine (LPC) levels as an example; Figure S3: Principal component analyses based on the overall lipidomic data of the comparison Sepsis-2 versus Sepsis-3 patients (left) and patients with and without bacteremia; Figure S4: Subclassification of sepsis patients based on lipid metabolites that differed significantly when comparing patients with and without bacteremia; Figure S5: The sphingolipid profile of patients with sepsis; Figure S6: The lipidomic profile of patients with sepsis; a unsupervised hierarchical clustering analysis based on triacyglygerol (TAG) metabolites that differed significantly between sepsis patients with and without bacteremia; Additional methodological information: Description of methodological strategies used by Metabolon for lipidomic analysis of patient samples.
