# Supplementary material for: Patients with Bacterial Sepsis Are Heterogeneous with Regard to Their Systemic Lipidomic Profiles
Source: Metabolites. 2022 Dec 29;13(1):52. doi: 10.3390/metabo13010052 (PMC9864715; doi:10.3390/metabo13010052)
Supplement: Supplementary file 1 [file metabolites-13-00052-s001.zip › metabolites-2063526-supplementary.pdf]

## Supplementary information

# Patients with bacterial sepsis are heterogeneous with regard to their systemic lipidomic profiles

**Knut Anders Mosevoll, Bent Are Hansen, Ingunn Margareetta Gundersen, Håkon Reikvam, Øyvind Bruserud, Øystein Bruserud and Øystein Wendelbo**

**Table S1:** Clinical characteristics of patients with and without bacteremia/blood stream infection (BSI); **Table S2:** Clinical and biological characteristics of patients with Gram-positive and Gram-negative infections; **Table S3:** The characteristics of individual patients included in the present study; **Table S4:** The selection of patients for the present study; **Table S5:** Differences of individual lipid metabolites between patients fulfilling the Sepsis-3 definition (PMID 26903338) versus patients only fulfilling the Sepsis-2 criteria (PMID 12682500, 12664219). **Table S6.** Differences of individual lipid metabolite concentrations between patients fulfilling the Sepsis-3 definition (PMID 26903338) versus patients only fulfilling the Sepsis-2 criteria (PMID 12682500, 12664219); **Table S7:** Differences of individual lipid metabolites between patients with and without bacterema; **Table S8:** Differences of individual lipid metabolites between patients with Gram-negative and Gram-positive infection; **Table S9.** Benjamini-Hochberg analyses of significantly different metabolites identified by the comparison of Sepsis-3 versus Sepsis-2 patients (upper part), and patients with and without bacteremia (lower part); **Figure S1:** The selection of patients for the present study; **Figure S2:** The metabolic variation of sepsis patients; total lysophosphatidylcholine (LPC) levels as an example; **Figure S3:** Principal component analyses based on the overall lipidomic data of the comparison Sepsis-2 versus Sepsis-3 patients (left) and patients with and without bacteriemia; **Figure S4:** Subclassification of sepsis patients based on lipid metabolites that differed significantly when comparing patients with and without bacteremia; **Figure S5:** The sphingolipid profile of patients with sepsis; **Figure S6.** The lipidomic profile of patients with sepsis; a unsupervised hierarchical clustering analysis based on triacyglygerol (TAG) metabolites that differed significantly between sepsis patients with and without bacteremia.; **Additional methodological information:** Description of methodological strategies used by Metabolon for lipidomic analysis of patient samples.

**Table S1.** Clinical characteristics of patients with and without bacteremia/blood stream infection (BSI).

| | *BSI (n=30)* | | *No BSI (n=30)* | | *p-value* |
|---|---|---|---|---|---|
| | *Median* | *Min/max* | *Median* | *Min/max* | |
| Age (years)[a] | 69.5 | 32-96 | 60 | 20-84 | **0.035** |
| Routine biochemistry[a] | | | | | |
| Hgb (g/dl) | 12.9 | 38.7-15.4 | 13 | 9.2-17.5 | 0.270 |
| WBC ($10^9$/L) | 13.4 | 5.1-27.9 | 14.7 | 4.9-46.6 | 0.318 |
| Neutrophils ($10^9$/L) | 11.3 | 3.4-26.6 | 12.1 | 4.1-41.1 | 0.587 |
| CRP(mg/L) | 177 | 4-457 | 160 | 7-538 | 0.196 |
| Erythrocyte sedimentation rate (ESR) | 52 | 19-107 | 34 | 5-126 | 0.101 |
| Creatinine (umol/L) | 108 | 48-706 | 72 | 27-475 | 0.015 |
| Temperature (°C) | 38.8 | 36.5-41.4 | 38.4 | 36.3-41.5 | 0.908 |
| Clinical findings at hospitalization[a] | | | | | |
| Heart rate (/min) | 108 | 70-140 | 113 | 75-136 | 0.505 |
| Respiratory frequency (/min) | 24 | 15-60 | 24 | 14-53 | 0.908 |
| Systolic blood pressure (mmHg) | 124 | 72-180 | 126 | 86-160 | 0.596 |
| Diastolic blood pressure (mmHg) | 67 | 26-110 | 50 | 50-104 | **0.003** |
| Glascow coma scale (GCS) | 15 | 3-15 | 15 | 14-15 | 0.169 |
| Lowest systolic blood pressure (mmHg) | 90 | 65-124 | 100 | 65-347 | 0.176 |
| Lowest middle artery pressure (MAP) | 61 | 50-105 | 50 | 50-96 | 0.495 |
| PaO2 (kPa) | 8.6 | 6.4-13.6 | 9.0 | 4.6-17.9 | 0.893 |
| FiO2 | 0.21 | 0.21-0.33 | 0.21 | 0.21-0.42 | 0.985 |
| PaO2/FiO2 | 41 | 21-54 | 41 | 11-85 | 0.825 |
| Organ failure[b,c] | | | | | |
| Total SOFA≥2 | 25 | 83% | 10 | 33% | |
| Any failure SOFA≥2 | 21 | 70% | 6 | 20% | |
| Respiratory failure | 13 | 77% | 12 | 40 % | **0.004** |
| Hypotension | 19 | 63% | 12 | 40 % | 0.071 |
| Bleeding disorder | 10 | 33% | 3 | 10% | **0.028** |
| Renal failure | 13 | 43% | 4 | 13% | **0.010** |
| Liver failure | 6 | 20% | 5 | 17% | 0.739 |
| CNS failure | 5 | 18% | 3 | 10% | 0.448 |
| | | | | | |
| Infection site[b] | | | | | **0.030** |
| Urinary tract | 15 | 50% | 13 | 43% | |
| Respiratory | 3 | 10% | 6 | 20% | |
| Soft tissue | 3 | 10% | 8 | 27% | |
| CNS | 3 | 10% | 0 | 0 | |
| Endocarditis | 5 | 17% | 0 | 0 | |
| Other | 1 | 3% | 3 | 10% | |

[a] *Median (min–max), Mann–Whitney U test.*
[b] *N(%), $\chi^2$ test.*
[c] *Defined as increase in sequential (sepsis-related) organ failure assessment score ≥2, sepsis induced (pre-existing, stable organ failure not included).*
[d] *Significant values are displayed in bold.*

**Table S2.** Clinical and biological characteristics of patients with Gram-positive and Gram-negative infections.

| | *Gram-positive (n=30)* | | *Gram-negative(n=30)* | | *p-value*[d] |
|---|---|---|---|---|---|
| | *Median* | *Range* | *Median* | *Range* | |
| Age (years)[a] | 61 | 20-96 | 73.5 | 30-91 | **0.043** |
| **Routine biochemistry**[a] | | | | | |
| Hgb (g/dl) | 13.1 | 8.7-17.5 | 12.7 | 9.8-15.9 | 0.286 |
| WBC ($10^9$/L) | 13.4 | 4.9-46.6 | 15.1 | 5.1-27.9 | 0.795 |
| Neutrophils ($10^9$/L) | 11.3 | 4.1-41.1 | 12.9 | 3.4-25.4 | 0.782 |
| CRP(mg/L) | 166 | 7-538 | 171 | 4-335 | 0.801 |
| Erythrocyte sedimentation rate (ESR) | 36 | 5-126 | 53 | 24-92 | **0.038** |
| Creatinine (umol/L) | 80 | 46-706 | 103 | 27-186 | 0.890 |
| Temperature (°C) | 38.8 | 36.3-40.9 | 38.5 | 36.5-41.5 | 0.863 |
| **Clinical findings at hospitalization**[a] | | | | | |
| Heart rate (/min) | 113 | 70-140 | 106 | 74-131 | 0.138 |
| Respiratory frequency (/min) | 24 | 14-60 | 24 | 15-40 | 0.597 |
| Systolic blood pressure (mmHg) | 128 | 79-180 | 122 | 11-153 | 0.238 |
| Diastolic blood pressure (mmHg) | 77 | 39-110 | 68 | 26-97 | 0.081 |
| Glascow coma scale (GCS) | 15 | 3-15 | 15 | 13-15 | 0.185 |
| Lowest systolic blood pressure (mmHg) | 92 | 65-134 | 100 | 73-126 | 0.173 |
| Lowest middle artery pressure (MAP) | 64 | 50-105 | 59 | 50-93 | 0.722 |
| PaO2 (kPa) | 8.7 | 4.6-17.9 | 6.4 | 6.4-15.2 | 0.940 |
| FiO2 | 0.21 | 0.21-0.42 | 0.21 | 0.21-0.27 | 0.880 |
| PaO2/FiO2 | 38 | 11-85 | 41 | 26-72 | 0.312 |
| Organ failure[b.c] | | | | | |
| Total SOFA≥2 | 22 | 73% | 13 | 43% | **0.018** |
| Any failure SOFA≥2 | 17 | 57% | 10 | 33% | 0.069 |
| Respiratory failure | 23 | 77% | 12 | 40% | **0.004** |
| Hypotension | 19 | 63% | 12 | 40% | 0.071 |
| Bleeding disorder | 8 | 27% | 5 | 17% | 0.347 |
| Renal failure | 8 | 27% | 9 | 30% | 0.774 |
| Liver failure | 5 | 17% | 6 | 20% | 0.739 |
| CNS failure | 7 | 23% | 1 | 3% | **0.023** |
| **Infection site**[b] | | | | | |
| Urinary tract | 2 | 7% | 26 | 87% | <0.00001 |
| Respiratory | 8 | 27% | 1 | 3% | 0.0257 |
| Soft tissue | 11 | 37% | 0 | 0 | 0.0003 |
| CNS | 3 | 10% | 0 | 0 | Ns |
| Endocarditis | 5 | 17% | 0 | 0 | Ns |
| Other | 1 | 3% | 3 | 10% | Ns |

[a] *Median (min–max). Mann–Whitney U test.*
[b] *N(%). $\chi^2$ test.*
[c] *Defined as increase in sequential (sepsis-related) organ failure assessment score ≥2, sepsis induced (pre-existing, stable organ failure not included).*
[d] *Significant values are displayed in bold.*

**Table S3.** The characteristics of individual patients included in the present study. We included 164 consecutive patients that fulfilled the Sepsis-2 criteria, in a previous clinical study (PMID 31934319). Four patients were immunocompromized at the time of admittance, and 80 patients did not have bacterial infections. Thus, 80 immunocompetent patients had documented bacterial infections and fulfilled the Sepsis-2 criteria (PMID 12682500, 12664219). Twenty of these 80 patients were excluded because they had mixed infections (i.e. two infecting bacteria detected, five patients), exceptional bacterial etiology (six patients), only detection of bacterial antigen (i.e. indirect evidence) but no bacterial growth in any patient samples (eight patients), and one randomly selected patient with Gram-negative infection;we then had two equal groups of Gram-positive and Gram-negative infections. Two random patients with pneumococcal infections were still included in our study in order to keep the number balance of Gram-positive/Gram-negative infections. The Sepsis-3 criteria (PMID 26903338) were fulfilled for 35 of the 60 patients. The number to the left in the table is the order of inclusion of the 80 patients with proven bacteriological etiology.

| **Included patients, number is order in the inclusion.** | **Microbe, blood culture** | **Microbe, other medium** |
|---|---|---|
| 1 | | *Escherichia coli* (urine) |
| 2 | *Staphylococcus aureus* | *Staphylococcus aureus* (tissue) |
| 3 | | *Escherichia coli* (urine) |
| 4 | | *Streptococcus pyogenes/Staphylococcus aureus* (Skin) |
| 5 | | *Staphylococcus saprophyticus* (urine) |
| 6 | *Escherichia coli* | *Escherichia coli* (urine) |
| 7 | *Escherichia coli* | *Escherichia coli* (urine) |
| 8 | *Streptococcus pneumoniae* | *Streptococcus pneumoniae* (positive urine antigen) |
| 9 | | *Streptococcus pneumoniae* (positive urine antigen) |
| 11 | *Escherichia coli* | *Escherichia coli* (urine) |
| 13 | | *Escherichia coli* (urine) |
| 14 | *Escherichia coli* | *Escherichia coli* (urine) |
| 16 | *Staphylococcus aureus* | |
| 17 | | *Streptococcus pyogenes* (soft tissue) |
| 19 | | *Escherichia coli* (urine) |
| 22 | *Streptococcus pyogenes* | |
| 23 | | *Escherichia coli* (urine) |
| 24 | | *Streptococcus pneumonia* and *Streptococcus milleri* in sterile brush from lung |
| 25 | *Group C streptococcus* | |
| 28 | | *Staphylococcus aureus* (wound secretion) |
| 30 | *Escherichia coli* | |
| 32 | | *Staphylococcus epidermidis* (wound and prosthesis) |
| 33 | *Staphylococcus aureus* | |
| 34 | | *Streptococcus pyogenes* (skin abscess) |
| 35 | *Staphylococcus aureus* | |
| 36 | *Staphylococcus aureus* | |
| 38 | *Escherichia coli (ESBL)* | |
| 39 | | *Escherichia coli* (urine) |
| 40 | *Escherichia coli (ESBL)* | |
| 41 | *Staphylococcus aureus* | |
| 42 | | *Streptococcus agalactia* (urine) |
| 43 | *Escherichia coli* | *Escherichia coli* (urine) |
| 44 | *Streptococcus pneumoniae* | |
| 46 | | *Streptococcus pyogenes* (tissue) |
| 47 | | *Escherichia coli* (urine) |
| 48 | *Streptococcus pneumoniae* | |
| 49 | | *Escherichia coli* (urine) |
| 50 | *Escherichia coli* | |
| 51 | | *Staphylococcus aureus* |
| 53 | | *Escherichia coli* (urine) |
| 60 | *Staphylococcus aureus* | *Staphylococcus aureus* (tracheal secret) |
| 61 | | *Serratia marcescens* (urine) |
| 62 | *Streptococcus pneumoniae* | *Streptococcus pneumoniae* (spinal fluid) |
| 63 | *Escherichia coli* | *Escherichia coli* (urine) |
| 64 | *Streptococcus pneumoniae* | *Streptococcus pneumoniae* (spinal fluid) |

| | | |
|---|---|---|
| 65 | *Escherichia coli* | *Escherichia coli* (urine) |
| 66 | | *Streptococcus pneumoniae* (positive urine antigen) |
| 67 | *Escherichia coli* | |
| 68 | | *Escherichia coli* (urine) |
| 69 | *Escherichia coli* | *Escherichia coli* (urine) |
| 70 | | *Escherichia coli* (urine) |
| 71 | | *Streptococcus intermedius* (pleural fluid) |
| 72 | | *Streptococcus dysglaciate* |
| 73 | *Escherichia coli* | |
| 74 | | *Escherichia coli* (urine) |
| 75 | *Streptococcus intermedius* | |
| 76 | | *Streptococcus pyogenes* |
| 77 | *Escherichia coli* | *Escherichia coli* (urine) |
| 78 | | *Escherichia coli*, ESBL (urine) |
| 79 | | *Proteus mirabilis* (urine) |

**Table S4.** The selection of patients for the present study. The table gives a list of excluded patients compared with our previous clinical study. We included 164 consecutive patients that fulfilled the Sepsis-2 criteria, in a previous clinical study (PMID 31934319). Four patients were immunocompromized at the time of admittance, and 80 patients did not have bacterial ifections. Thus, 80 immunocompetent patients had documented bacterial infections and fulfilled the Sepsis-2 criteria (PMID 12682500, 12664219). Twenty of these 80 patients were excluded because they had mixed infections (i.e. two infecting bacteria detected, five patients), exceptional bacterial etiology (six patients), only detection of bacterial antigen but no bacterial growth in any patient samples, and one randomly selected patient with Gram-negative infection; we then had two equal groups of Gram-positive and Gram-negative infections. The Sepsis-3 criteria (PMID 26903338) were fulfilled for 35 of the 60 patients.

| **Excluded patients, order of inclusion.** | **Microbe, blood culture** | **Microbe, other medium** |
|---|---|---|
| 10 | | *Streptococcus pneumoniae* (positive urine antigen) |
| 12 | | *Streptococcus pneumoniae* (positive urine antigen) |
| 15 | *Klebsiella pneumoniae* | *Klebsiella pneumoniae* and *Escherichia coli* (urine) |
| 18 | *Klebsiella pneumoniae* and *Proteus mirabilis* | Streptococcus pneumoniae (positive urine antigen) |
| 20 | | *Acinubaculum schalii* (urine) |
| 21 | *Enterobacter cloacae* | |
| 26 | *Bacteroides fragilis* | |
| 27 | | *Streptococcus pneumoniae* (positive urine antigen) |
| 29 | | Clostridium toxin A |
| 31 | *Fusobacterium necroforum* | |
| 37 | *Streptococcus constellatus* and *Actinobaculum schaalii* | |
| 45 | *Kingella kingae* | |
| 52 | *Neisseria meningitidis* | |
| 54 | | *Streptococcus pneumoniae* (positive urine antigen) |
| 55 | *Klebciella pneumoniae* | |
| 56 | | *Streptococcus pneumoniae* (positive urine antigen) |
| 57 | *Streptococcus pneumoniae* | *Escherichia coli* (urine) |
| 58 | *Bacteroides ovatus* and *uniformis* | |
| 59 | | *Streptococcus pneumoniae* (positive urine antigen) |
| 80 | *Escherichia coli* | |

**Table S5.** Differences of individual lipid metabolites between patients fulfilling the Sepsis-3 definition (PMID 26903338) versus patients only fulfilling the Sepsis-2 criteria (PMID 12682500, 12664219). All 15 individual metabolites showing a significant difference in ANOVA analysis are listed in the table. The 15 metabolites are ranged according to their p-value. The table presents the classification (Main pathway/subpathway), identity, ratio of mean levels (Sepsis-3 level relative to Sepsis-2 level) and the corresponding p-value. A ratio >1.00 is indicated by red (i.e. higher in Sepsis-3 patients) whereas a ratio <1.00 is indicated by green. Metabolites showing detectable levels for less than 10 patients are marked with *.

| Main pathway | Subpathway | Metabolite | Sepsis-3 versus Sepsis-2 ratio | p-value |
|---|---|---|---|---|
| * Phosphatidylethanolamine | PE Ether | PE(O-18:0/18:0) | 1.13 | 0.0000 |
| Sphingolipids | Dihydroceramide | DCER(22:1) | 1.47 | 0.0049 |
| Sphingolipids | Dihydroceramide | DCER(20:1) | 1.71 | 0.0123 |
| Diacylglycerol | DAG Ester | DAG(14:1/18:1) | 1.56 | 0.0176 |
| Sphingolipids | Dihydroceramide | DCER(18:1) | 1.50 | 0.0242 |
| Sphingolipids | Lactosylceramide | LCER(16:0) | 0.76 | 0.0243 |
| * Phosphatidylinositol | PI Ester | PI(18:1/16:1) | 0.84 | 0.0258 |
| Sphingolipids | Sphingomyelin | SM(24:0) | 0.80 | 0.0318 |
| Sphingolipids | Ceramide | CER(24:0) | 0.79 | 0.0385 |
| Phosphatidylinositol | PI Ester | PI(18:0/18:2) | 0.75 | 0.0400 |
| Sphingolipids | Lactosylceramide | LCER(20:0) | 0.86 | 0.0414 |
| Sphingolipids | Sphingomyelin | SM(18:1) | 0.78 | 0.0425 |
| Phosphatidylethanolamine | PE Plasmalogen | PE(O-18:1/16:0) | 1.46 | 0.0450 |
| Cholesterol Ester | CE Ester | CE(20:2) | 0.73 | 0.0452 |
| Diacylglycerol | DAG Ester | DAG(14:0/18:3) | 1.26 | 0.0476 |

**Table S6.** Differences of individual lipid metabolite concentrations between patients fulfilling the Sepsis-3 definition (PMID 26903338) versus patients only fulfilling the Sepsis-2 criteria (PMID 12682500, 12664219). All 15 individual metabolites showing a significant difference in ANOVA analysis are listed in the figure. The 13 metabolites are ranged according to their p-value. The table presents the classification (Main pathway/subpathway), identity, the absolute level (median and range) for Sepsis-3 and Sepsis-2 level patients and the corresponding p-value. One metabolite was left out because less than 10 patients showed detectable levels. All concentrations are presented in µM. PE(O-18:0/18:0) and PI(18:1/16:1) were detected in less than 10 patients and were not included in the table. Increased and decreased levels in Sepsis-3 patients are indicated by red and green color, respectively.

| Subpathway | Metabolite | Sepsis-3 versus Sepsis-2 | Sepsis 3 patients Metabolite concentration | Sepsis 2 patients Metabolite concentration | p-value |
|---|---|---|---|---|---|
| Dihydroceramide | DCER(22:1) | | 2.46 (1.38-5.15) | 2,34 (1,703,75) | 0.0049 |
| Dihydroceramide | DCER(20:1) | | 0.88 (0.53-2.27) | 0.90 (0.44-2.48) | 0.0123 |
| DAG Ester | DAG(14:1/18:1) | | 0.48 (0,30-3.31) | 0.51 (0.22-2.61) | 0.0176 |
| Dihydroceramide | DCER(18:1) | | 0.83 (0.38-1.93) | 0.77 (0.48-1.43) | 0.0242 |
| Lactosylceramide | LCER(16:0) | | 62.5 (49.6-77.5) | 60.9 (53.4-66.8) | 0.0243 |
| Sphingomyelin | SM(24:0) | | 5.10 (3.76-7,92) | 4.90 (3.01-7.51) | 0.0318 |
| Ceramide | CER(24:0) | | 36.5 (24.5-56.8) | 36.8 (24.7-56.2) | 0.0385 |
| PI Ester | PI(18:0/18:2) | | 17.0 (10.1-44.1) | 16,4 (10.1-21.8) | 0.0400 |
| Lactosylceramide | LCER(20:0) | | 1.87 (0.82-2.17) | 1,36 (0.77-2,35) | 0.0414 |
| Sphingomyelin | SM(18:1) | | 2,54 (1.88-3,65) | 2.56 (1.43-3.48) | 0.0425 |
| PE Plasmalogen | PE(P-18:1/16:0) | | 0.12 (0.05-0.19) | 0.11 (0.04-0.29) | 0.0450 |
| CE Ester | CE(20:2) | | 0.12 (0.07-0.25) | 0.11 (0.07-0.20) | 0.0452 |
| DAG Ester | DAG(14:0/18:3) | | 0.11 (0.03-0.29) | 0.10 (0.02-0.21) | 0.0476 |

**Table S7.** Differences of individual lipid metabolites between patients with and without bacterema. The 43 metabolites are listed according to their p-value. The table presents the classification (Main pathway/subpathway), identity, ratio of mean levels (with relative to without bacteremia level) and the corresponding p-value. A ratio >1.00 is indicated by red (i.e. higher in patients with bacteremia patients) whereas a ratio <1.00 is indicated by green. Metabolites showing detectable levels for less than 10 patients are marked by *.

| Main pathway | Subpathway | Metabolite | No bacteremia versus bacteremia ratio | p-value |
|---|---|---|---|---|
| * Phosphatidylethanolamine | PE Ether | PE(O-18:0/18:0) | 1.13 | 0.0000 |
| Triacylglycerol | TAG Ester | TAG55:6-FA20:3 | 0.61 | 0.0049 |
| * Lysophosphatidylcholine | LPC Ester | LPC(18:3) | 1.59 | 0.0084 |
| * Phosphatidylethanolamine | PE Ester | PE(18:1/22:0) | 1.55 | 0.0087 |
| Phosphatidylethanolamine | PE Plasmalogen | PE(P-18:1/18:1) | 1.47 | 0.0106 |
| Phosphatidylcholine | PC Ester | PC(18:2/20:4) | 2.07 | 0.0109 |
| Phosphatidylcholine | PC Ester | PC(18:2/20:3) | 1.75 | 0.0109 |
| Lysophosphatidylcholine | LPC Ester | LPC(18:1) | 1.96 | 0.0117 |
| Lysophosphatidylcholine | LPC Ester | LPC(18:2) | 2.16 | 0.0142 |
| Triacylglycerol | TAG Ester | TAG56:5-FA20:2 | 0.59 | 0.0146 |
| Phosphatidylethanolamine | PE Plasmalogen | PE(P-18:1/16:0) | 1.52 | 0.0156 |
| Triacylglycerol | TAG Ester | TAG56:4-FA20:1 | 0.60 | 0.0185 |
| Triacylglycerol | TAG Ester | TAG56:4-FA20:2 | 0.65 | 0.0196 |
| * Phosphatidylethanolamine | PE Plasmalogen | PE(P-18:2/22:6) | 1.21 | 0.0212 |
| Lysophosphatidylcholine | LPC Ester | LPC(16:1) | 1.77 | 0.0212 |
| Triacylglycerol | TAG Ester | TAG56:4-FA18:2 | 0.61 | 0.0232 |
| Triacylglycerol | TAG Ester | TAG54:3-FA18:0 | 0.67 | 0.0240 |
| Lysophosphatidylcholine | LPC Ester | LPC(20:3) | 2.16 | 0.0242 |
| Lysophosphatidylcholine | LPC Ester | LPC(15:0) | 1.52 | 0.0264 |
| Triacylglycerol | TAG Ester | TAG56:5-FA18:2 | 0.61 | 0.0278 |
| Triacylglycerol | TAG Ester | TAG54:3-FA18:2 | 0.68 | 0.0281 |
| Lysophosphatidylcholine | LPC Ester | LPC(17:0) | 1.70 | 0.0288 |
| Lysophosphatidylcholine | LPC Ester | LPC(14:0) | 1.32 | 0.0300 |
| Triacylglycerol | TAG Ester | TAG56:4-FA18:1 | 0.68 | 0.0304 |
| Lysophosphatidylcholine | LPC Ester | LPC(22:6) | 1.69 | 0.0336 |
| Lysophosphatidylcholine | LPC Ester | LPC(16:0) | 1.79 | 0.0353 |
| Lysophosphatidylethanolamine | LPE Ester | LPE(18:1) | 1.47 | 0.0359 |
| Sphingolipids | Lactosylceramide | LCER(18:0) | 0.84 | 0.0392 |
| Triacylglycerol | TAG Ester | TAG54:4-FA18:0 | 0.65 | 0.0396 |
| Triacylglycerol | TAG Ester | TAG55:4-FA18:2 | 0.66 | 0.0397 |
| Diacylglycerol | DAG Ester | DAG(14:0/18:3) | 1.64 | 0.0398 |
| Triacylglycerol | TAG Ester | TAG56:3-FA20:1 | 0.64 | 0.0403 |
| Triacylglycerol | TAG Ester | TAG56:5-FA20:1 | 0.61 | 0.0410 |
| Lysophosphatidylcholine | LPC Ester | LPC(18:0) | 1.78 | 0.0428 |
| Triacylglycerol | TAG Ester | TAG42:0-FA14:0 | 1.26 | 0.0433 |
| Sphingolipids | Dihydroceramide | DCER(22:1) | 1.34 | 0.0434 |
| Diacylglycerol | DAG Ester | DAG(14:0/20:0) | 1.44 | 0.0435 |
| Triacylglycerol | TAG Ester | TAG55:8-FA20:4 | 0.62 | 0.0443 |
| Sphingolipids | Dihydroceramide | DCER(20:1) | 1.71 | 0.0453 |

| | | | | |
|---|---|---|---|---|
| Triacylglycerol | TAG Ester | TAG40:0-FA14:0 | **1.03** | 0.0461 |
| Triacylglycerol | TAG Ester | TAG54:4-FA18:2 | **0.70** | 0.0474 |
| Triacylglycerol | TAG Ester | TAG44:0-FA14:0 | **1.42** | 0.0478 |
| * Lysophosphatidylcholine | LPC Ester | LPC(20:0) | **1.37** | 0.0493 |

The triglycerides utilize the following nomenclature: TAG(A):(B)-FA(C).
A: The total number of carbons present within the three acyl chains esterified to the glycerol backbone of the triacylglycerol (TAG) molecule.
B: The total number of double bonds amongst the three acyl chains esterified to the glycerol backbone.
C: The carbon length and saturation status of one of the three acyl chains.
The TAG species TAG36:0-FA12:0 represents an example. From the measurement one knows that the three acyl chains associated with the molecule have a total of 36 carbon atoms and that none of these acyl chains are desaturated (as indicated by the TAG36:0 designation). One also knows that at least one of the triacylglycerols/TAG's acyl chains is 12 carbons long (as indicated by the FA12:0 designation). The length of the two other acyl chains is not specified, but one knows that they must have a sum equal to 24 (e.g., 36-12=24).

**Table S8.** Differences of individual lipid metabolites between patients with Gram-negative and Gram-positive infection. The metabolites are listed according to their p-value. The table presents the classification (Main pathway/subpathway), identity, ratio of mean levels (Gram-negative versus Gram-positive infection) and the corresponding p-value. A ratio >1.00 is indicated by red (i.e. higher in patients with Gram-negative infection) whereas a ratio <1.00 is indicated by green. Metabolites showing detectable levels for less than 10 patients are marked by *.

| Main pathway | Subpathway | Metabolite | Gram-negative versus Gram-positive ratio | p-value |
|---|---|---|---|---|
| * Phosphatidylethanolamine | PE Ether | PE(O-18:0/18:0) | 1.13 | 0.0000 |
| Sphingolipids | Hexosylceramide | HCER(26:1) | 1.41 | 0.0006 |
| Phosphatidylethanolamine | PE Plasmalogen | PE(P-18:1/20:5) | 3.05 | 0.0076 |
| Phosphatidylethanolamine | PE Plasmalogen | PE(P-18:0/20:5) | 2.54 | 0.0102 |
| Phosphatidylethanolamine | PE Plasmalogen | PE(P-16:0/20:5) | 2.33 | 0.0149 |
| * Phosphatidylinositol | PI Ester | PI(18:1/16:1) | 0.84 | 0.0170 |
| * Phosphatidylethanolamine | PE Plasmalogen | PE(P-18:2/22:6) | 1.23 | 0.0233 |
| Diacylglycerol | DAG Ester | DAG(14:0/22:6) | 1.63 | 0.0272 |
| * Phosphatidylcholine | PC Ester | PC(16:0/14:1) | 0.74 | 0.0280 |
| Diacylglycerol | DAG Ester | DAG(18:0/22:6) | 1.58 | 0.0307 |
| Diacylglycerol | DAG Ester | DAG(16:0/22:6) | 1.73 | 0.0323 |
| Phosphatidylethanolamine | PE Ether | PE(O-18:0/20:5) | 1.71 | 0.0338 |
| Monoacylglycerol | Ester | MAG(18:4) | 1.40 | 0.0339 |
| Phosphatidylcholine | PC Ester | PC(18:0/22:4) | 0.68 | 0.0370 |
| Phosphatidylethanolamine | PE Plasmalogen | PE(P-18:1/22:6) | 1.47 | 0.0412 |
| Sphingolipids | Lactosylceramide | LCER(26:1) | 1.37 | 0.0413 |
| Diacylglycerol | DAG Ester | DAG(18:2/22:6) | 1.67 | 0.0457 |
| Phosphatidylethanolamine | PE Plasmalogen | PE(P-18:1/16:0) | 1.53 | 0.0474 |
| * Phosphatidylcholine | PC Ester | PC(20:0/22:6) | 1.19 | 0.0484 |
| * Phosphatidylethanolamine | PE Ether | PE(O-16:0/20:5) | 1,53 | 0.0485 |
| * Lysophosphatidylethanolamine | LPE Ester | LPE(18:3) | 0.42 | 0.0498 |

**Table S9.** Benjamini-Hochberg analyses of significantly different metabolites identified by the comparison of Sepsis-3 versus Sepsis-2 patients (upper part), and patients with and without bacteremia (lower part). The table presents only the five metabolites with the highest ranking based on the corresponding p-values. The presentation includes the biochemical identity, identity, ratio of mean levels (Sepsis 3 versus Sepsis2, patients with versus without bacteremia), the p and q values from the statistical comparison, ranking of biochemicals/metabolites based on the p-value, number of comparisons (i.e. identified biochemical) and the Gram-negative versus Gram-positive infection) and the calculated Benjamini-Hochberg critical value for each of these biochemicals/metabolites. This value bas based on a false discovery rate of 0.25. The metabolite being significant after this analysis is marked with blue.

| SEPSIS-3 | | | | | | |
|---|---|---|---|---|---|---|
| **Biochemical** | **Sepsis 3/2** | **p value** | **q value** | **p ranking** | **Number of comparisons** | **(l/m)Q** |
| PE(O-18:0/18:0) | **1.13** | 0.0000 | 0.0000 | 1 | 966 | 0.0002588 |
| DCER(22:1) | **1.47** | 0.0049 | 0.9938 | 2 | 966 | 0.0005176 |
| DCER(20:1) | **1.71** | 0.0123 | 0.9938 | 3 | 966 | 0.0007764 |
| DAG(14:1/18:1) | **1.56** | 0.0176 | 0.9938 | 4 | 966 | 0.0010352 |
| DCER(18:1) | **1.50** | 0.0242 | 0.9938 | 5 | 966 | .001294 |
| **BACTEREMIA** | | | | | | |
| **Biochemical** | **Bakteremi+/-** | **p value** | **q value** | **p ranking** | **Number of comparisons** | **(l/m)Q** |
| PE(O-18:0/18:0) | **1.13** | 0.0000 | 0.0000 | 1 | 966 | 0.0002588 |
| TAG55:6-FA20:3 | **0.61** | 0.0049 | 0.7769 | 2 | 966 | 0.0005176 |
| LPC(18:3) | **1.59** | 0.0084 | 0.7769 | 3 | 966 | 0.0007764 |
| PE(18:1/22:0) | **1.55** | 0.0087 | 0.7769 | 4 | 966 | 0.0010352 |
| PE(P-18:1/18:1) | **1.47** | 0.0106 | 0.7769 | 5 | 966 | 0.001294 |

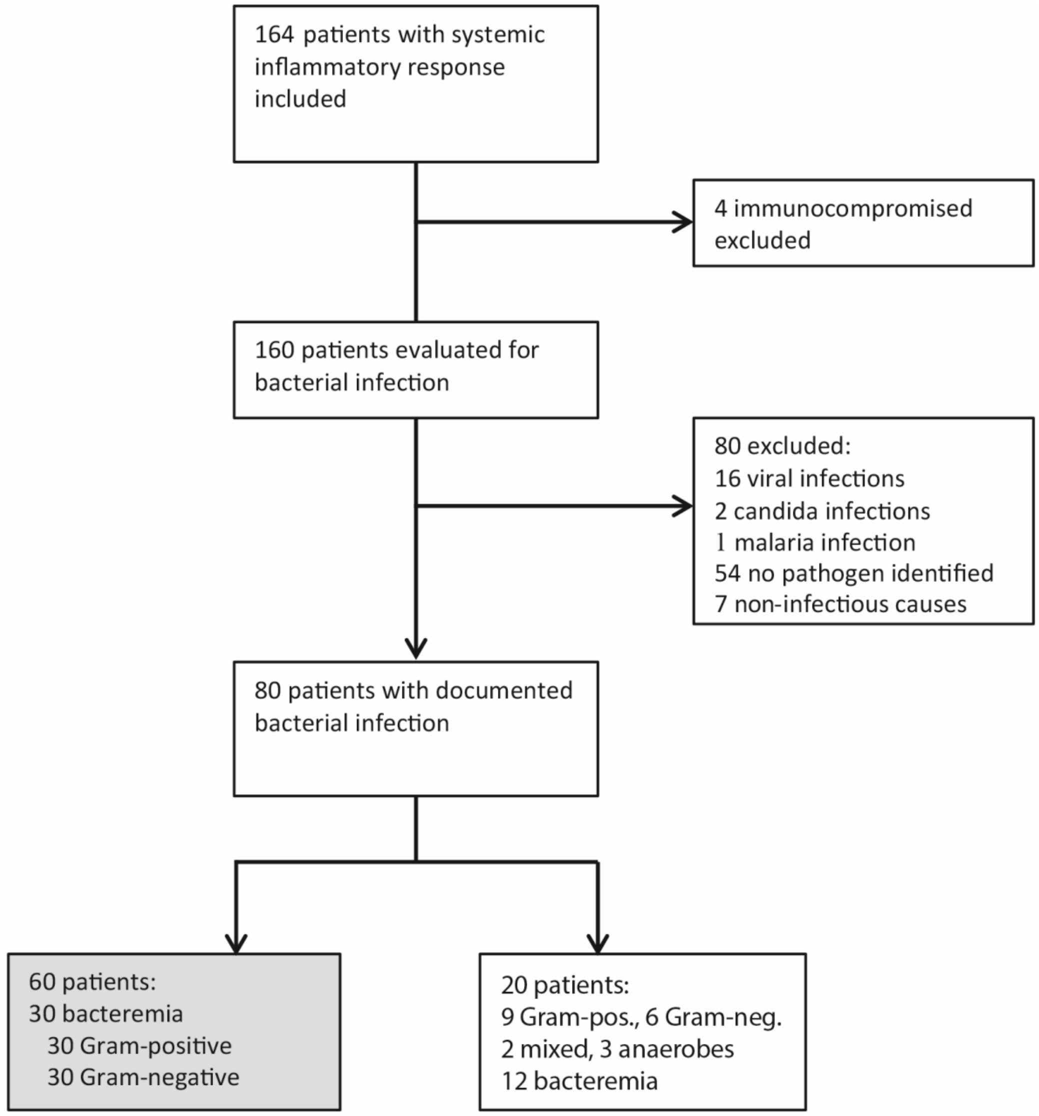


**Figure S1**. The selection of patients for the present study. We included 164 consecutive patients that fulfilled the Sepsis-2 criteria, in a previous clinical study (PMID 31934319). Four patients were immunocompromized at the time of admittance, and 80 patients did not have bacterial ifections. Thus, 80 immunocompetent patients had documented bacterial infections and fulfilled the Sepsis-2 criteria (PMID 12682500, 12664219). Twenty of these 80 patients were excluded because they had mixed infections (i.e. two infecting bacteria detected, five patients), exceptional bacterial etiology (six patients), only detection of bacterial antigen but no bacterial growth in any patient samples, and one randomly selected patient with Gram-negative infection; we then had two equal groups of Gram-positive and Gram-negative infections. The Sepsis-3 criteria (PMID 26903338) were fulfilled for 35 of the 60 patients.

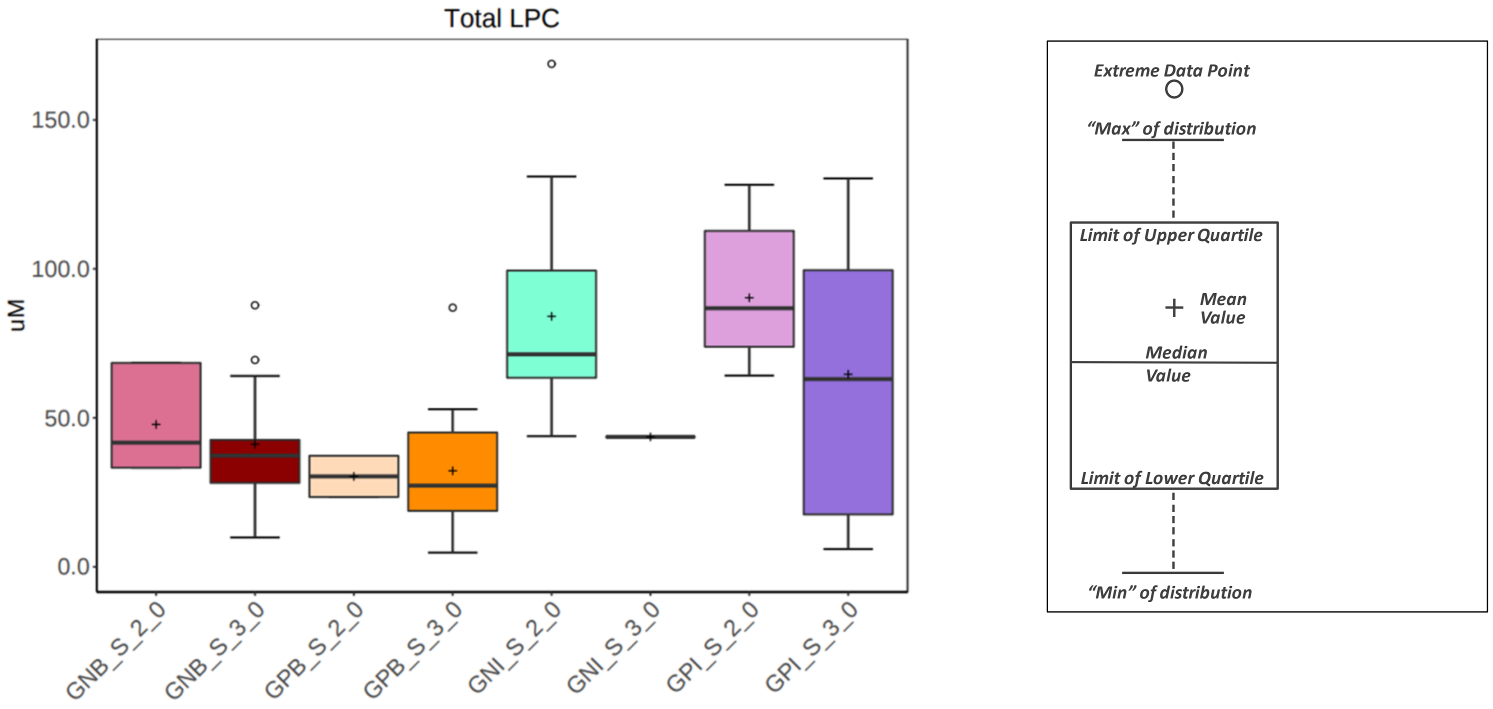


**Figure S2.** The metabolic variation of sepsis patients; total lysophosphatidylcholine (LPC) levels as an example showing the levels/range of the measured metabolite concentrations in various patient subsets. The meaning of the boxes/whiskers are explained in the right figure. The serum levels (μM) are presented for the subsets from left to right for the following subsets (left part of the figure):
GNB_S_2_0: Gram-negative infections, patients with bacteremia, only fulfilling Sepsis-2 citeria;
GNB_S_3_0: Gram-negative infections, patients with bacteremia, fulfilling Sepsis-3 citeria;
GPB_S_2_0: Gram-positive infections, patients with bacteremia, only fulfilling Sepsis-2 citeria GPB_S_3_0: Gram-positive infections, patients with bacteremia, fulfilling Sepsis-3 citeria;
GNI_S_2_0: Gram-negative infections, no bacteremia, only fulfilling Sepsis-2 citeria;
GNI_S_3_0: Gram-negative infections, no bacteremia, fullfiling Sepsis-3 citeria;
GPI_S_2_0: Gram-positive infections, no bacteremia, only fulfilling Sepsis-2 citeria;
GPI_S_3_0: Gram-positive infections, no bacteremia, fulfilling Sepsis-3 citeria.

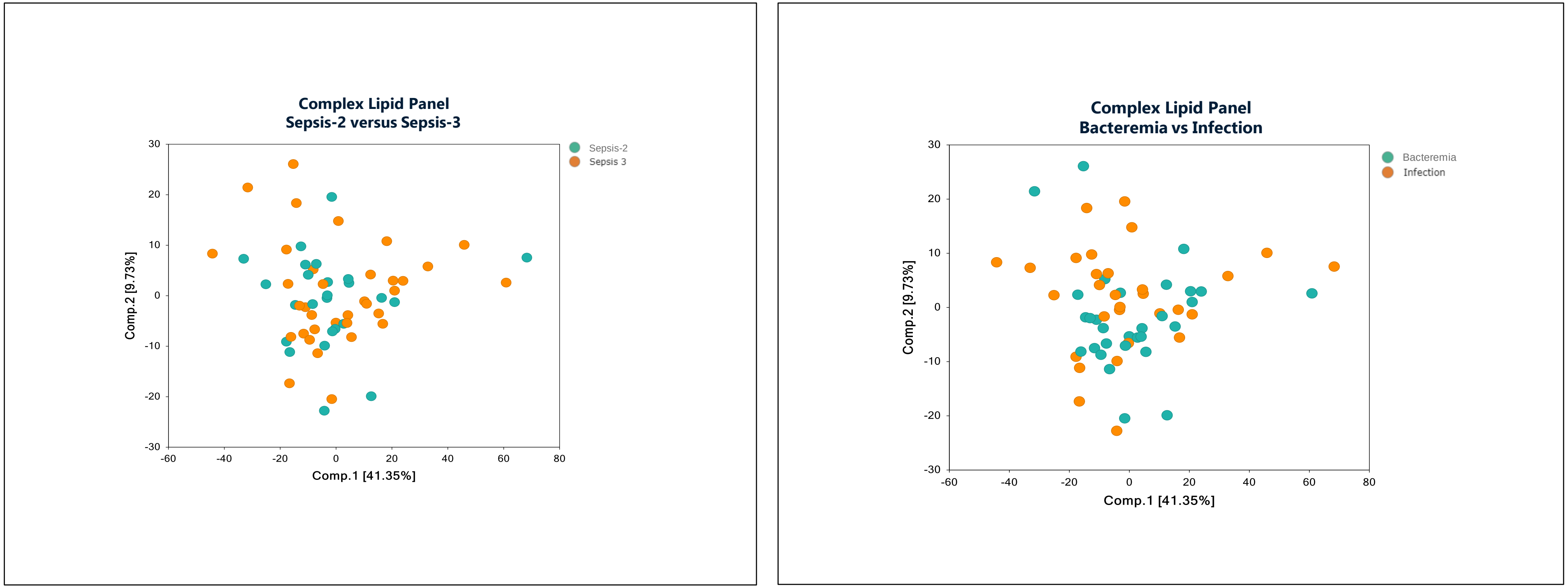


**Figure S3.** Principal component analyses based on the overall lipidomic data of the comparison Sepsis-2 versus Sepsis-3 patients (left) and patients with and without bacteriemia. The contribution of each component to the variation is shown on the axes.

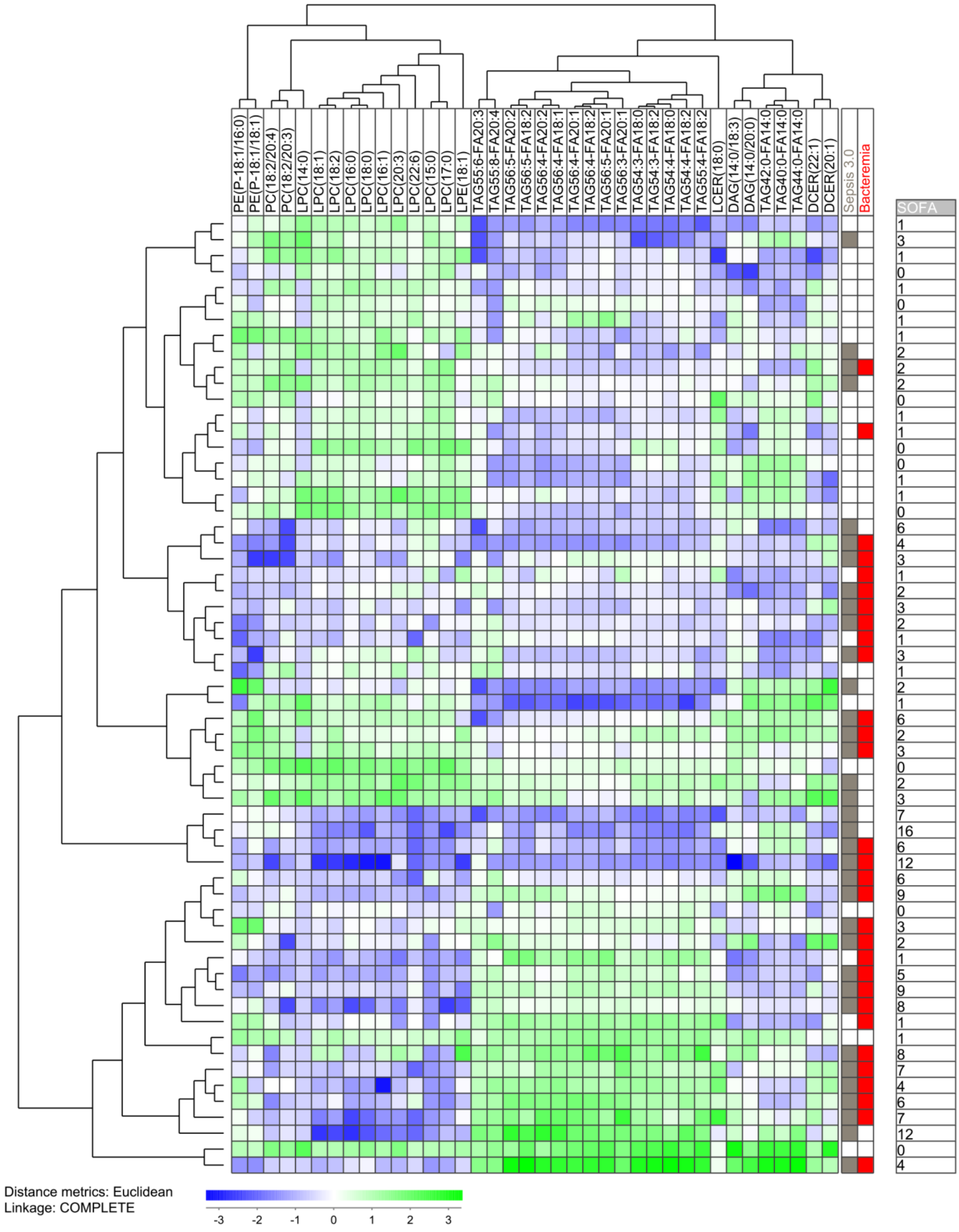


**Figure S4.** Subclassification of sepsis patients based on lipid metabolites that differed significantly when comparing patients with and without bacteremia. We performed an unsupervised hierarchical clustering analysis based on the 38 (out of 43) metabolites that reached detectable levels for at least 10 patients The characteristics of each individual patient (fulfilling Sepsis-3 criteria, detection of bacteremia, total SOFA score) are indicated to the right in the figure. Abbreviations; PE, phosphatidylethanolamine; PC, phsphatidylcholine; LPC, lysophosphatidylcholine; LPE, lysophosphatidylethanolamine; TAG, triacylglycerol; LCER, lactosylceramide; DAG, diacylglycerol; DCER, dihydroceramide.

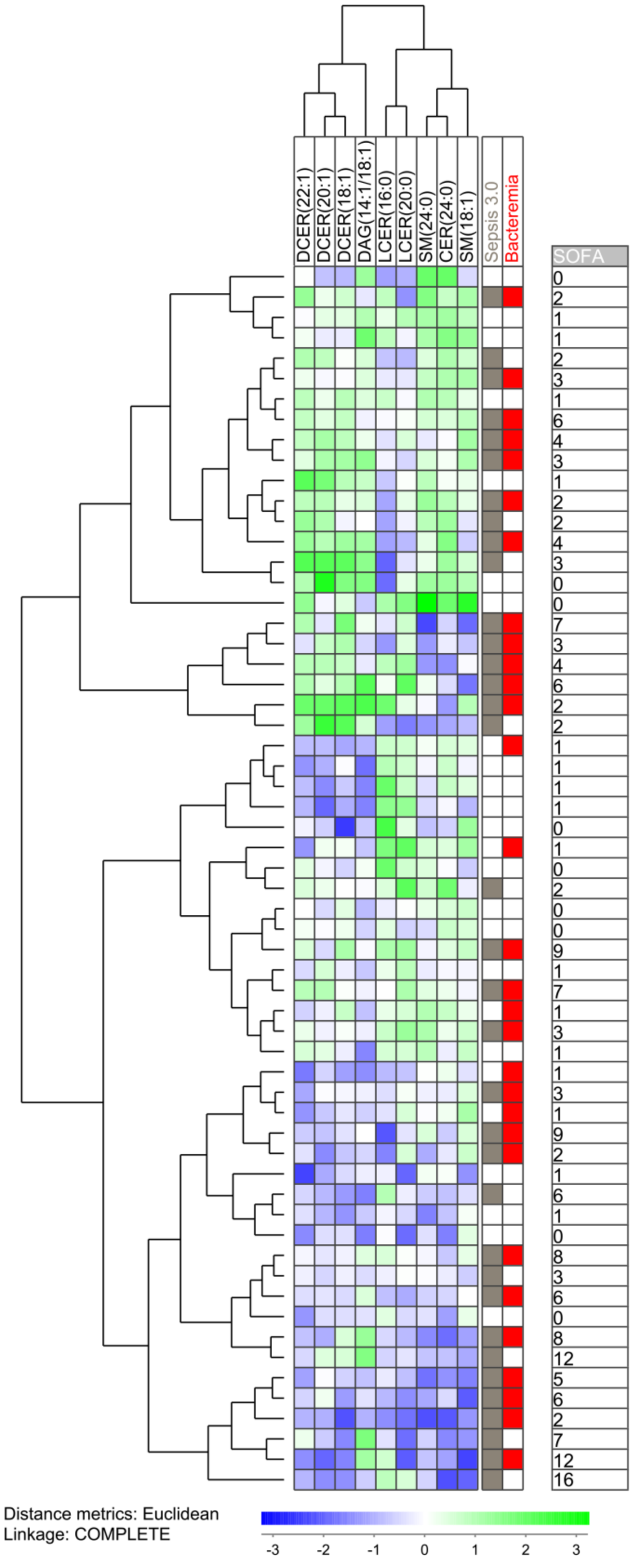


**Figure S5.** The sphingolipid profile of patients with sepsis. We performed an unsupervised hierarchical clustering analysis including all 60 patients and based on eight sphingolipid metabolites (i) showing detectable levels for at least 10 patients and (ii) showing a statistically significant difference between Sepsis-3 and Sepsis-2 patients. The characteristics of each individual patient (organ failure, bacteremia, total SOFA score) are indicated to the right in the figure. Abbreviations: DCER, dihydroceramides; LCER, lactosylceramides, SM, sphingomyelin; CE, cholesterol ester; CER, ceramide; PI, phsphatidylinositol.

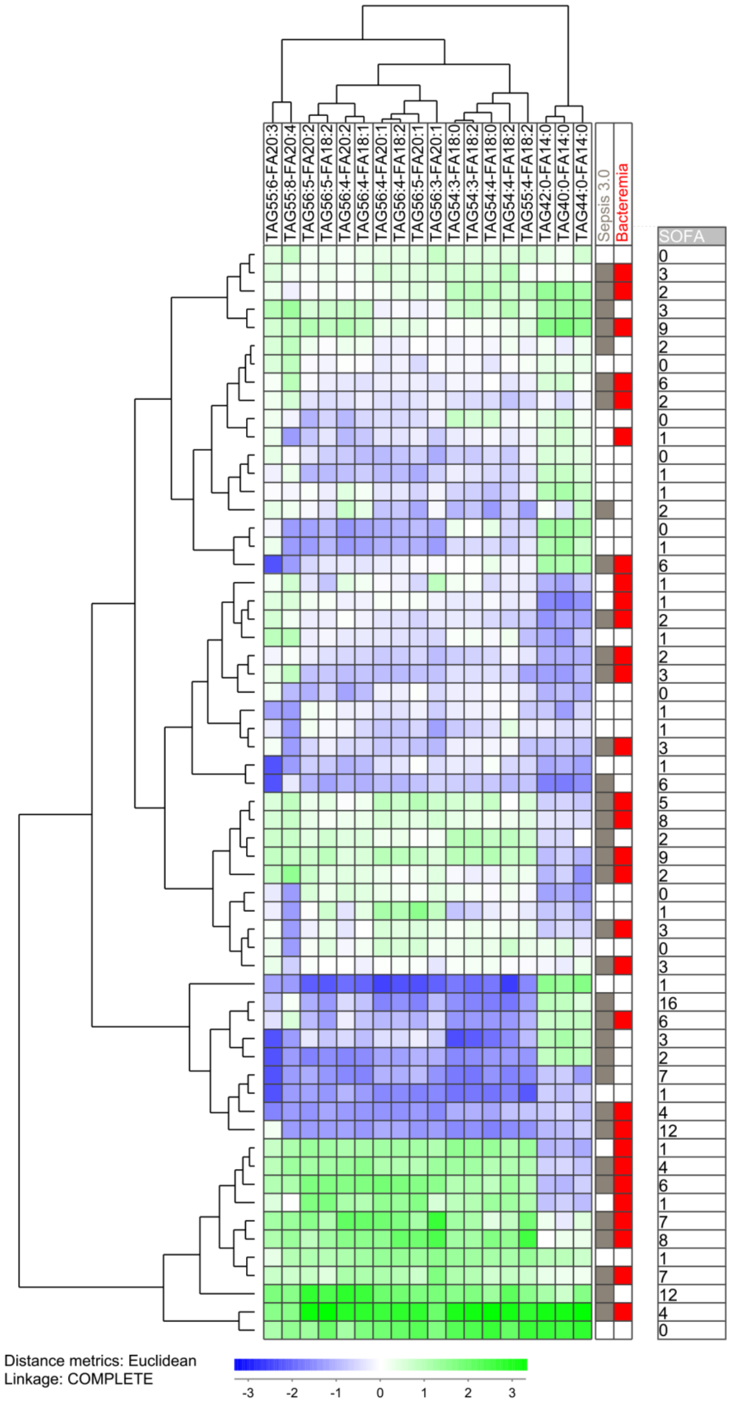


**Figure S6.** The lipidomic profile of patients with sepsis; a unsupervised hierarchical clustering analysis based on triacyglygerol (TAG) metabolites that differed significantly between sepsis patients with and without bacteremia. We performed an unsupervised hierarchical clustering analysis including all 60 patients and based on all 18 metabolites (i) showing detectable levels for at least 10 patients and (ii) showing a statistically significant difference when comparing patients fulfilling the Sepsis-3 criteria versus patients only fulfilling the Sepsis-2 criteria (Sepsis-3 versus Sepsis-2 patient levels). The characteristics of each individual patient (Sepsis-3 classification, detection of bacteremia, total SOFA score) are presented to the right in the figure. Abbreviations: PE,phsphatidyletanolamine ; DAG, diacyglycerols; DCER, dihydroceramides; LCER, lactosylceramides, SM, sphingomyelin; CE, cholesterol ester; CER, ceramide; PI, phsphatidylinositol.

**ADDITIONAL METHODOLOGICAL INFORMATION**
**DESCRIPTION OF METHODOLOGICAL STRATEGIES USED BY METABOLON FOR LIPIDOMIC ANALYSIS OF PATIENT SAMPLES**

## *Metabolon Platform*

**Sample Accessioning:** Following receipt, samples were inventoried and immediately stored at -80°C. Each sample received was accessioned into the Metabolon LIMS system and was assigned by the LIMS a unique identifier that was associated with the original source identifier only. This identifier was used to track all sample handling, tasks, results, etc. The samples (and all derived aliquots) were tracked by the LIMS system. All portions of any sample were automatically assigned their own unique identifiers by the LIMS when a new task was created; the relationship of these samples was also tracked. All samples were maintained at -80°C until processed.

**Sample Preparation:** Samples were prepared using the automated MicroLab STAR® system from Hamilton Company. Several recovery standards were added prior to the first step in the extraction process for QC purposes. To remove protein, dissociate small molecules bound to protein or trapped in the precipitated protein matrix, and to recover chemically diverse metabolites, proteins were precipitated with methanol under vigorous shaking for 2 min (Glen Mills GenoGrinder 2000) followed by centrifugation. The resulting extract was divided into five fractions: two for analysis by two separate reverse phase (RP)/UPLC-MS/MS methods with positive ion mode electrospray ionization (ESI), one for analysis by RP/UPLC-MS/MS with negative ion mode ESI, one for analysis by HILIC/UPLC-MS/MS with negative ion mode ESI, and one sample was reserved for backup. Samples were placed briefly on a TurboVap® (Zymark) to remove the organic solvent. The sample extracts were stored overnight under nitrogen before preparation for analysis.

**QA/QC:** Several types of controls were analyzed in concert with the experimental samples: a pooled matrix sample generated by taking a small volume of each experimental sample (or alternatively, use of a pool of well-characterized human plasma) served as a technical replicate throughout the data set; extracted water samples served as process blanks; and a cocktail of QC standards that were carefully chosen not to interfere with the measurement of endogenous compounds were spiked into every analyzed sample, allowed instrument performance monitoring and aided chromatographic alignment. Tables S10 and S11 describe these QC samples and standards. Instrument variability was determined by calculating the median relative standard deviation (RSD) for the standards that were added to each sample prior to injection into the mass spectrometers. Overall process variability was determined by calculating the median RSD for all endogenous metabolites (i.e., non-instrument standards) present in 100% of the pooled matrix samples. Experimental samples were randomized across the platform run with QC samples spaced evenly among the injections, as outlined in Figure S7.

**Table S10: Description of Metabolon QC Samples**

| Type | Description | Purpose |
|---|---|---|
| MTRX | Large pool of human plasma maintained by Metabolon that has been characterized extensively. | Assure that all aspects of the Metabolon process are operating within specifications. |
| CMTRX | Pool created by taking a small aliquot from every customer sample. | Assess the effect of a non-plasma matrix on the Metabolon process and distinguish biological variability from process variability. |
| PRCS | Aliquot of ultra-pure water | Process Blank used to assess the contribution to compound signals from the process. |
| SOLV | Aliquot of solvents used in extraction. | Solvent Blank used to segregate contamination sources in the extraction. |

**Table S11: Metabolon QC Standards**

| Type | Description | Purpose |
|---|---|---|
| RS | Recovery Standard | Assess variability and verify performance of extraction and instrumentation. |
| IS | Internal Standard | Assess variability and performance of instrument. |

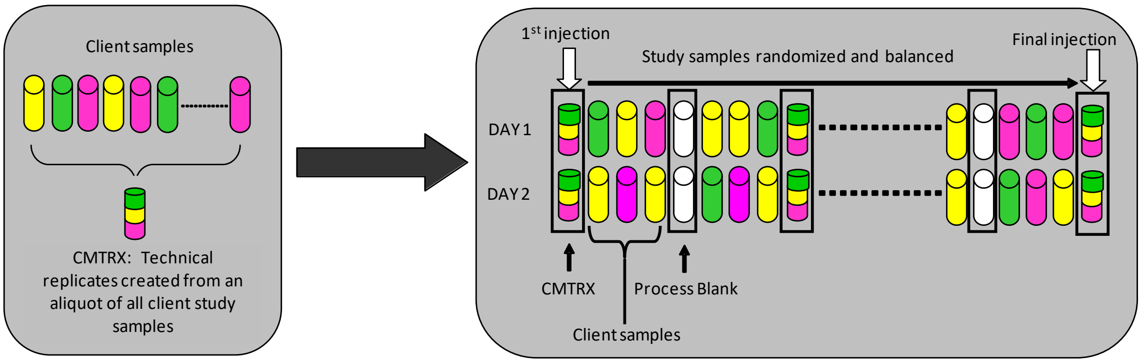


**Figure S7. Preparation of client-specific technical replicates.** A small aliquot of each client sample (colored cylinders) is pooled to create a CMTRX technical replicate sample (multi-colored cylinder), which is then injected periodically throughout the platform run. Variability among consistently detected biochemicals can be used to calculate an estimate of overall process and platform variability.

**Ultrahigh Performance Liquid Chromatography-Tandem Mass Spectroscopy (UPLC-MS/MS):** All methods utilized a Waters ACQUITY ultra-performance liquid chromatography (UPLC) and a Thermo Scientific Q-Exactive high resolution/accurate mass spectrometer interfaced with a heated electrospray ionization (HESI-II) source and Orbitrap mass analyzer operated at 35,000 mass resolution. The sample extract was dried then reconstituted in solvents compatible to each of the four methods. Each reconstitution solvent contained a series of standards at fixed concentrations to ensure injection and chromatographic consistency. One aliquot was analyzed using acidic positive ion conditions, chromatographically optimized for more hydrophilic compounds. In this method, the extract was gradient eluted from a C18 column (Waters UPLC BEH C18-2.1x100 mm, 1.7 µm) using water and methanol, containing 0.05% perfluoropentanoic acid (PFPA) and 0.1% formic acid (FA). Another aliquot was also analyzed using acidic positive ion conditions, however it was chromatographically optimized for more hydrophobic compounds. In this method, the extract was gradient eluted from the same afore mentioned C18 column using methanol, acetonitrile, water, 0.05% PFPA and 0.01% FA and was operated at an overall higher organic content. Another aliquot was analyzed using basic negative ion optimized conditions using a separate dedicated C18 column. The basic extracts were gradient eluted from the column using methanol and water, however with 6.5mM Ammonium Bicarbonate at pH 8. The fourth aliquot was analyzed via negative ionization following elution from a HILIC column (Waters UPLC BEH Amide 2.1x150 mm, 1.7 µm) using a gradient consisting of water and acetonitrile with 10mM Ammonium Formate, pH 10.8. The MS analysis alternated between MS and data-dependent $MS^n$ scans using dynamic exclusion. The scan range varied slighted between methods but covered 70-1000 m/z. Raw data files are archived and extracted as described below.

**Bioinformatics:** The informatics system consisted of four major components, the Laboratory Information Management System (LIMS), the data extraction and peak-identification software, data processing tools for QC and compound identification, and a collection of information interpretation and visualization tools for use by data analysts. The hardware and software foundations for these informatics components were the LAN backbone, and a database server running Oracle 10.2.0.1 Enterprise Edition.

**LIMS:** The purpose of the Metabolon LIMS system was to enable fully auditable laboratory automation through a secure, easy to use, and highly specialized system. The scope of the Metabolon LIMS system encompasses sample accessioning, sample preparation and instrumental analysis and reporting and advanced data analysis. All of the subsequent software systems are grounded in the LIMS data structures. It has been modified to leverage and interface with the in-house information extraction and data visualization systems, as well as third party instrumentation and data analysis software.

**Data Extraction and Compound Identification:** Raw data was extracted, peak-identified and QC processed using Metabolon's hardware and software. These systems are built on a web-service platform utilizing Microsoft's .NET technologies, which run on high-performance application servers and fiber-channel storage arrays in clusters to provide active failover and load-balancing. Compounds were identified by comparison to library entries of purified standards or recurrent unknown entities. Metabolon maintains a library based on authenticated standards that contains the retention time/index (RI), mass to charge ratio (*m/z)*, and chromatographic data (including MS/MS spectral data) on all molecules present in the library. Furthermore, biochemical identifications are based on three criteria: retention index within a narrow RI window of the proposed identification, accurate mass match to the library +/- 10 ppm, and the MS/MS forward and reverse scores between the experimental data and authentic standards. The MS/MS scores are based on a comparison of the ions present in the experimental spectrum to the ions present in the library spectrum. While there may be similarities between these molecules based on one of these factors, the use of all three data points can be utilized to distinguish and differentiate biochemicals. More than 3300 commercially available purified standard compounds have been acquired and registered into LIMS for analysis on all platforms for determination of their analytical characteristics. Additional mass spectral entries have been created for structurally unnamed biochemicals, which have been identified by virtue of their recurrent nature (both chromatographic and mass spectral). These compounds have the potential to be identified by future acquisition of a matching purified standard or by classical structural analysis.

**Curation:** A variety of curation procedures were carried out to ensure that a high quality data set was made available for statistical analysis and data interpretation. The QC and curation processes were designed to ensure accurate and consistent identification of true chemical entities, and to remove those representing system artifacts, mis-assignments, and background noise. Metabolon data analysts use proprietary visualization and interpretation software to confirm the consistency of peak identification among the various samples. Library matches for each compound were checked for each sample and corrected if necessary.

**Metabolite Quantification and Data Normalization:** Peaks were quantified using area-under-the-curve. For studies spanning multiple days, a data normalization step was performed to correct variation resulting from instrument inter-day tuning differences. Essentially, each compound was corrected in run-day blocks by registering the medians to equal one (1.00) and normalizing each data point proportionately (termed the "block correction"; Figure 2). For studies that did not require more than one day of analysis, no normalization is necessary, other than for purposes of data visualization. In certain instances, biochemical data may have been normalized to an additional factor (e.g., cell counts, total protein as determined by Bradford assay, osmolality, etc.) to account for differences in metabolite levels due to differences in the amount of material present in each sample.

## *Complex Lipid Platform*

Lipids were extracted from the serum in the presence of deuterated internal standards using an automated BUME extraction according to the method of Lofgren et al. (J Lipid Res 2012:53(8):1690-

700). The extracts were concentrated under nitrogen and reconstituted in 0.25mL of 10mM ammonium acetate dichloromethane:methanol (50:50). The extracts were transferred to inserts and placed in vials for infusion-MS analysis, performed on a Shimazdu LC with nano PEEK tubing and the Sciex SelexIon-5500 QTRAP. The samples were analyzed via both positive and negative mode electrospray. The 5500 QTRAP scan was performed in MRM mode with the total of more than 1,100 MRMs. Individual lipid species were quantified by taking the peak area ratios of target compounds and their assigned internal standards, then multiplying by the concentration of internal standard added to the sample. Lipid species concentrations were background-subtracted using the concentrations detected in process blanks (water extracts) and run day normalized (when applicable). The resulting background-subtracted, run-day normalized lipid species concentrations were then used to calculate the lipid class and fatty acid total concentrations, as well as the mol% composition values for lipid species, lipid classes, and fatty acids.
